# Machine and Deep Learning for Detection of Moderate-to-Vigorous Physical Activity From Accelerometer Data: Systematic Scoping Review

**DOI:** 10.2196/76601

**Published:** 2026-01-08

**Authors:** Yahua Zi, Sjors RB van de Ven, Eco JC de Geus, Peijie Chen

**Affiliations:** 1 School of Exercise and Health Shanghai University of Sport Shanghai China; 2 Department of Biological Psychology Faculty of Behavioural and Movement Sciences Vrije Universiteit Amsterdam Amsterdam The Netherlands

**Keywords:** physical activity intensity, raw accelerometer data, wearable sensors, free-living validation, classification, estimation, sensor placement, machine learning, deep learning

## Abstract

**Background:**

Accurate monitoring of moderate-to-vigorous physical activity (MVPA) is critical for advancing public health research and personalized interventions. Traditional accelerometry methods, reliant on regression-derived intensity cut points, exhibit significant misclassification errors and poor generalizability to the free-living environment. Recent advancements in machine learning (ML) and deep learning (DL) offer promising alternatives for automated MVPA detection.

**Objective:**

This scoping review synthesizes evidence on ML and DL techniques for MVPA estimation and prediction using accelerometer data, focusing on performance, algorithm bias, sensor configurations, and translational potential.

**Methods:**

Following PRISMA-ScR (Preferred Reporting Items for Systematic Reviews and Meta-Analyses Extension for Scoping Reviews) guidelines, we conducted a systematic search across PubMed, IEEE Xplore, and Web of Science (February 1995-April 2025), supplemented by snowball citation tracking. Two independent reviewers screened titles, abstracts, and full texts against predefined inclusion criteria. Data from included studies were charted by one reviewer and verified by the other, extracting details on study characteristics, sensor configuration, ML and DL techniques, validation methods, and performance metrics. A narrative synthesis approach was used, guided by 6 research questions, to collate and summarize the findings. The synthesis process was rigorously reviewed by multiple authors to ensure consistency.

**Results:**

Of 1938 screened studies, 40 met the inclusion criteria, with 4 studies added by follow-up manual searches. While traditional ML models (eg, random forest, support vector machine) achieved strong laboratory performance with *F*_1_-score of 87.4%-100% and accuracy of 87.9%-100%, their real-world performance declined by 8.0%-13.3% in *F*_1_-score and 6.6%-12.2% in accuracy, due to environment noise and device heterogeneity. DL architectures (eg, convolutional neural networks, transformers) achieved robust performance by leveraging raw signal dynamics with an *F*_1_-score of 71.9%-79.8% and an accuracy of 87.9%-100% in free-living settings. Hybrid models (eg, convolutional neural networks and long short-term memory) demonstrated state-of-the-art performance (*F*_1_-score 91.4%-98.4%, accuracy 97.7%-99.0%). Wrist-worn sensors dominated studies (30/40, 75%) and matched hip/thigh placements in lab settings (mean *F*_1_-scores: 86.5%-88.6%), but multisensor configurations (wrist + hip) yielded the highest accuracy (89.7%). Key challenges included algorithmic bias reducing applicability in older adult populations, and impaired reproducibility, with only 42.5% (17/40) of studies sharing code and data. Emerging opportunities are noted for edge computing and hybrid models integrating contextual data.

**Conclusions:**

ML and DL significantly enhance MVPA monitoring by automating feature extraction and improving adaptability to free-living variability. However, persistent gaps in generalizability, inconsistent validation protocols, and transparency deficits hinder translation. The findings support the need for future research to prioritize inclusive model training, standardized reporting frameworks, and open science practices to realize the equitable potential of artificial intelligence–driven physical activity assessment.

## Introduction

Moderate-to-vigorous physical activity (MVPA) is defined as activities requiring specific metabolic equivalent of tasks (METs), such as 3 METs or 4 METs [1,2]. It is critical to preventive health, linked to reduced risks of cardiovascular disease [3,4], diabetes [5], and premature mortality [6]. Current guidelines, such as those from the World Health Organization, emphasize MVPA as a priority; for example, children and adolescents are advised to engage in MVPA with an average of 60 minutes per day across the week to improve health [7,8]. Additionally, accurate measurement of physical activity is critical for identifying the individual, environmental, and sociocultural determinants and evaluating the efficacy of intervention strategies. Accelerometer-based motion sensors, owing to their compact design, durability, and low cost, have emerged as the predominant tool for objective physical activity assessment in diverse populations [9-12].

Traditional accelerometry methods, though widely adopted, have historically been underused in research due to reliance on intensity-based cut points derived from linear regression models or receiver operating curves [13,14]. These approaches establish thresholds by predicting energy expenditure from accelerometer counts. However, proprietary count-based thresholds, such as Freedson’s cut points [15], exhibit significant misclassification of activity intensity (eg, sedentary, light, moderate, vigorous intensity) of approximately 50% in adults [16] and 28%-45% in children and adolescents [17-20]. Such methods fail to account for biomechanical nuances (eg, energy expenditure differences between walking on flat terrain vs uphill terrain) or uncontrolled variables in free-living environments, such as nonexercise movements [21,22]. The proliferation of conflicting regression-derived cut points has further complicated cross-study comparisons [23]. While these thresholds remain standard for quantifying activity intensity, their inability to accurately predict intensity across diverse activities is increasingly acknowledged [22].

The advent of machine learning (ML) and deep learning (DL) has revolutionized intensity recognition by enabling feature extraction and classification from raw accelerometer signals [24,25]. Compared with traditional cut point methods, ML models (eg, random forests [RFs] and support vector machines [SVMs]) leverage time- and frequency-domain features from high-resolution triaxial data (eg, 30-100 Hz) to reduce energy expenditure errors by 25%-50% in school-age children [19,26]. More recently, DL architectures, such as a convolutional neural network (CNN) for local temporal pattern detection, a long short-term memory network (LSTM) for modeling activity sequences, Transformers for long-range dependency learning, and hybrid models (eg, convolutional neural network and long short-term memory [CNN-LSTM]), have further advanced the field. These models identify MVPA bouts by modeling temporal dependencies in continuous data streams [27].

Three distinct methodological approaches have emerged for MVPA detection: The first one is based on activity classification, which directly identifies MVPA from activity-specific movement patterns [28,29]. The second one is based on energy expenditure prediction from predefined MET thresholds (eg, ≥3 METs) [19,30]. The third one is based on an end-to-end DL architecture that automates hierarchical feature extraction from raw accelerometer signals to classify activity intensity directly or through energy expenditure estimation [31-33]. Hybrid models, such as CNN-LSTM, further enhance performance by integrating spatial feature extraction (via convolutions) with temporal modeling (via recurrent layers) to identify subtle biomechanical patterns (eg, stride variability during running) and contextual transitions between movements [34]. However, over 60% of models remain inaccessible due to unshared code or validation protocols, perpetuating a “new cut-point conundrum” that undermines cross-study comparability and clinical utility [35].

Other shortcomings further undermine progress in MVPA-specific research. First, lab-based findings fail to be generalized to real-world conditions. For example, RF achieves >90% accuracy in lab settings [36,37], but its free-living performance degrades dramatically to around 66% [38]. However, only 10% of studies validate models in the real world [39], limiting translational relevance. Second, disparities in validation protocols, such as settings (laboratory-controlled vs free-living environments), or device placement (hip vs wrist), complicate cross-study comparisons. For instance, models trained on hip-based ActiGraph data often underperform when applied to wrist-worn devices [40]. Third, ethical and reproducibility challenges, such as algorithmic bias against older adults or clinical cohorts, and limited code or data sharing, hinder the translation.

Several systematic reviews have explored the broader field of activity recognition using accelerometers and artificial intelligence (AI). However, a focused synthesis on AI-driven MVPA detection is lacking. Previous reviews have either focused on physical activity type detection in real-life conditions rather than intensity-specific thresholds [41], provided the general methodologies of human activity recognition using wearable sensors and ML without a systematic analysis of performance and bias in MVPA classification [42-44], examined the validation of accelerometer-based monitors using ML but not within the specific context of MVPA’s lab-to-real-world gap [27], or highlighted the critical issue of accessibility and reproducibility of novel analytical models but not connected them to the development of equitable MVPA models [35]. Other reviews have focused on specific aspects, such as calibration techniques [45], sport-specific movements [46], or compared DL architectures like CNN and LSTM [47]. While 2 recent reviews touch on predicting physical activity intensity from smartphones or smartwatches [48,49], they do not encompass the full spectrum of research-grade and wearable sensors, model architectures, and the critical synthesis of translational challenges presented here.

Therefore, this scoping review is the first to systematically scope and synthesize the literature exclusively on ML and DL techniques for MVPA intensity. We uniquely quantify the performance of MVPA detection methods as a function of the sensors used, sensor placement, target populations, feature extraction strategies, model architectures, lab-to-real-world settings, and look at possible algorithmic bias introduced by the restricted age and health status of the tested participants.

## Methods

### Overview

This scoping review follows the Arksey and O’Malley framework, which includes 5 key stages: identifying the research question (RQ), identifying relevant studies, selecting studies, charting the data, and collating, summarizing, and reporting the results. The PRISMA-ScR (Preferred Reporting Items for Systematic Reviews and Meta-Analyses extension for Scoping Reviews) was also consulted to ensure methodological rigor. EndNote X9 (Clarivate Analytics) was used for reference management, deduplication, and the screening process.

### Identify the Research Questions

This paper presents a scoping review that synthesizes advancements in ML- and DL-driven MVPA estimation and prediction from accelerometer data. The review aims to answer the following RQs:

- RQ1: What ML and DL techniques have been and are currently used for MVPA detection from accelerometer data?
- RQ2: How do accelerometer specifications (eg, sensor type, sampling rate), body placement (eg, wrist, hip, and thigh), and multisensor configurations influence model performance and generalizability?
- RQ3: What’s the magnitude of the performance gap between laboratory-controlled and free-living environments, and what potential factors contribute to this disparity?
- RQ4: How do validation protocols vary across studies, and how do inconsistencies in these protocols limit cross-study comparability and clinical utility?
- RQ5: To what extent do current models exhibit biased results, preventing generalization to older adult or clinical populations?
- RQ6: What proportion of studies adhere to open science practices, and how do transparency gaps hinder reproducibility, scalability, and equitable deployment?

### Identify Relevant Studies

To ensure a comprehensive and focused literature search, we used a multi-step process. The search strategy was developed and refined in discussion with all the authors, who have specialized expertise in systematic review methodologies and database search strategies. Initially, we conducted a preliminary manual search to identify eligible studies and determine relevant databases and query terms. The search strategy included the following keywords and their combinations: “artificial intelligence” (eg, “machine learning” and “deep learning”), “accelerometer” (eg, “wearable device,” “smartphone,” “smartwatch,” and “inertial measurement unit (IMU)”), and “moderate-to-vigorous physical activity” (eg, MVPA, “physical activity intensity,” and “energy expenditure”).

The comprehensive search was conducted across multiple databases, including PubMed, IEEE Xplore, and Web of Science. The search was conducted on April 4, 2025. To further enhance the comprehensiveness of our search, a manual citation search was conducted using reference lists of relevant studies to identify other potentially eligible studies. The detailed search strategies used to find relevant studies for this scoping review are described in Multimedia Appendix 1.

### Selection of Eligible Studies

We followed the steps outlined in the PRISMA (the Preferred Reporting Items for Systematic Reviews and Meta-Analyses extension guidelines) flow diagram to select eligible studies. The study selection process involved 2 independent reviewers (YZ and SRBVDV) who screened titles and abstracts, followed by full-text assessment. Any discrepancies were resolved through discussion. This process is illustrated in the PRISMA flow diagram. The inclusion criteria were as follows: (1) studies that applied ML or DL technique; (2) studies that used accelerometry, no matter any location, number of sensors, or any type of devices, such as accelerometers or smartphones or smartwatches; (3) studies that estimated of MVPA as the outcome; (4) studies that focused on human, any age group or any health status; and (5) peer-reviewed studies published in English.

The exclusion criteria were as follows: (1) studies that did not involve ML or DL techniques (2) studies that relied on multimodal sensor systems (eg, integrated heart rate monitors with accelerometers) or nonaccelerometric data (eg, video-based estimation); (3) studies that focused on nonhuman or nonphysical activity contexts, such as only differentiating sedentary behavior from nonsedentary activity; (4) studies that focused on general activity recognition or physical activity intensity classification without MVPA-specific analysis; (5) studies that focused on theoretical models without empirical validation; (6) studies that were not peer reviewed or reported in a non-English language; and (7) studies without full text available.

### Data Charting

Duplicates were identified and removed using the automated deduplication feature in EndNote X9, which was configured to define duplicates based on matching author, publication year, and title field. This automated process was followed by a manual check to ensure the thoroughness and accuracy of deduplication. Then, guided by the RQs, the following details were extracted from included studies: study characteristics (eg, author, publication year, population characteristics), sensor configuration (brand and model, placement, sampling rate), ML or DL techniques used to detect MVPA (features choosing strategy, features selected, MVPA classification technique), ground truth validation of MVPA (via indirect calorimetry [IC] or direct observation [DO]), validating setting (lab or free-living conditions), classification performance metrics (*F*_1_-score and accuracy), and code availability. In this review, only the *F*_1_-score and accuracy for MVPA classification were extracted. In cases where these metrics were not explicitly reported in the primary studies, they were inferred from the provided confusion matrices using the standard functions. Accuracy, representing the proportion of total correct predictions, was calculated as:

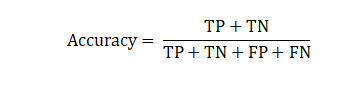



The *F*_1_-score, the harmonic mean of precision and recall, was calculated as

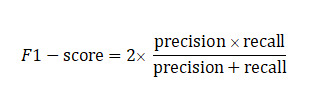



Where:

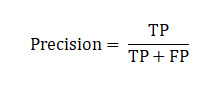

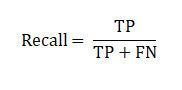





In which, TP is the number of true positives, TN is the number of true negatives, FP is the number of false positives, and FN is the number of false negatives.

### Collating, Summarizing, and Reporting the Results

To answer the RQs, the results are organized into 6 sections: evolution of feature engineering and model architectures, task-specific insights, sensor performance, validation practices, algorithmic bias, and reproducibility crisis.

A narrative synthesis approach was used, guided by the predefined RQs. The extracted data were summarized quantitatively (using frequencies and percentages) and qualitatively (identifying key themes and trends). To ensure rigor and trustworthiness, the data charting and initial synthesis were performed by one author (YZ) and critically reviewed by the others (SRBVDV, EJCDG, and PC) for accuracy and consistency.

### Ethical Considerations

This scoping review synthesized findings from previously published research involving human participants. No new participants were recruited, and no new primary data were collected for this review. Consequently, separate ethical approval for this specific synthesis was not required. All original studies included in this review were expected to have obtained appropriate ethical approval from their relevant institutional review boards or ethics committees and informed consent from participants, consistent with ethical standards for human participation research involving sensor data. We noted that the majority of included studies explicitly reported ethical approval within their publications. For studies where an explicit ethics statement was not found in the publication, we acknowledge this limitation in reporting transparency. As this review analyzed results reported in published literature and did not involve direct access to or reanalysis of the raw accelerometer data from the original studies, specific data licenses or permissions beyond the published findings were not required.

## Results

### Overview

**Figure 1:**
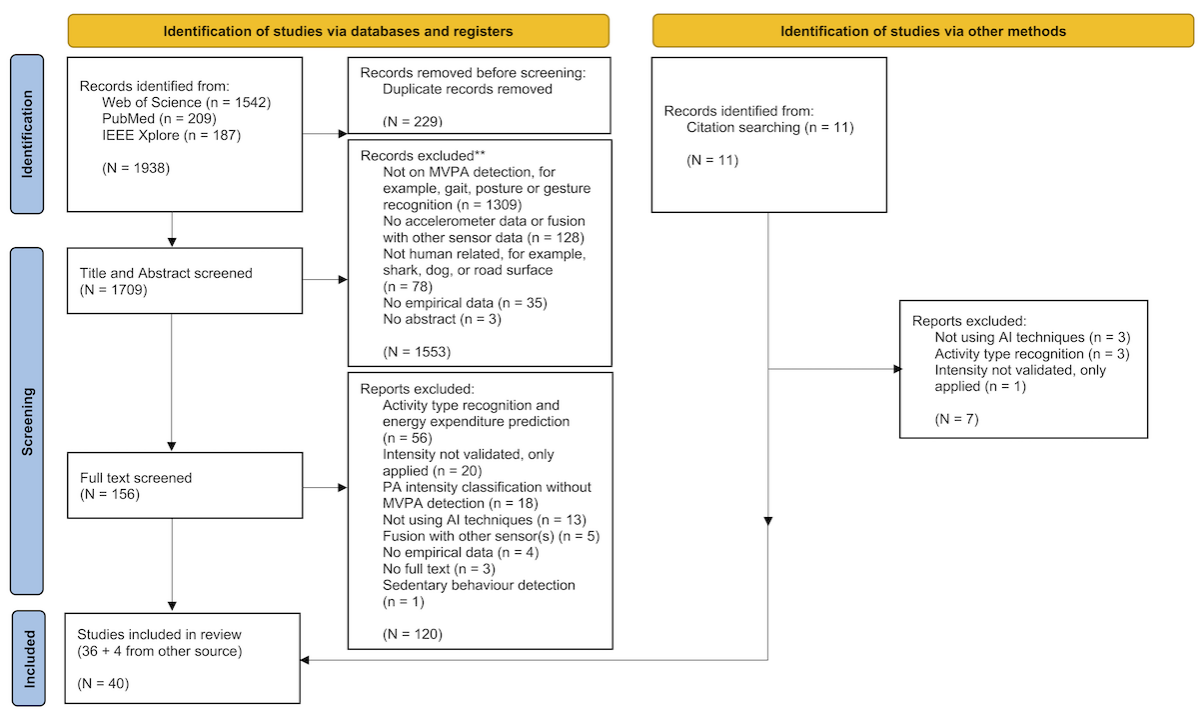
PRISMA flow diagram of study selection. AI: artificial intelligence; MVPA: moderate-to-vigorous physical activity; PA: physical activity.

A total of 1938 articles were identified from PubMed (n=209), IEEE Xplore (n=187), and Web of Science (n=1542). After removing 11.8% (229/1938) of duplicates, 88.2% (1709/1938) of the articles were included in the title and abstract screening phase. After this phase, 156 (8.1%) were screened for eligibility in the full-text screening phase. As a result, 36 articles met the inclusion criteria. In addition, 4 studies were included from manual searches. In total, 40 articles were included in this scoping review, shown in Figure 1.

### Overview of Included Studies

Table 1 provides a summary of included studies, including the author’s name and publication year, mean age of participants (SD), number of participants, sensor brand and specific model, number of sensors tested, the placement of sensors, MVPA classification techniques used, validation setting (lab or free-living), assessment of MVPA ground truth, and code availability. The information on the country where the test was conducted, sampling rate, feature choosing strategy, window length, feature selected, and *F*_1_-score and accuracy of model detecting MVPA was listed in Multimedia Appendix 2 [19,28,29,31,33,34,36-38,50-81].

**Table 1:** The summary of included studies (N=40 studies, ranked by health condition and alphabetically by author names).

Reference		Age (years), mean (SD)	Age (years), range	N	Sensor brand/ model	Number of sensors (placement)	MVPA^a^ classification technique	Validation setting	Ground truth	Code availability
**Healthy condition**										
	Ahmadi et al [50]	4 (0.9)	—^b^	31	ActiGraph GT3X +	2 (hip, wrist)	RF^c^ and SVM^d^	Free-living	DO^e^	No
	Ahmadi et al [51]	4 (0.9)	—	31	ActiGraph GT3X +	2 (hip, wrist)	RF	Free-living	DO	Yes
	Ahmadi et al [52]	13.9 (3)	—	50	ActiGraph GT3X +	1 (hip)	RF	Lab	PRE^f^	Yes
	Ahmadi et al [38]	4 (0.9)	—	31	ActiGraph GT3X +	2 (hip, wrist)	RF	Free-living	DO	Yes
	Ahmadi et al [53]	55.8 (12.4)	—	102	MEMS and ActiGraph GT9X	2 (both wrists)	RF	Lab and free-living	DO	No
	Ahmadi et al [53]	55.8 (12.4)	—	52	MEMS and ActiGraph GT9X	2 (both wrists)	RF	Lab and free-living	DO, IC^g^	No
	Ahmadi et al [53]	—	18-91	151	Axivity AX3	1 (wrist)	RF	Free-living	DO	No
	Andò et al [36]	NR^h^	—	NR	LSM9DS1	1 (chest)	k-NN^i^ and RF	Lab	PRE	No
	Bai et al [54]	72.4 (7.1)	—	247	ActiGraph GT3X-BT	1 (wrist)	XGBoost^j^	Lab	IC	No
	Barua et al [34]	29 (0)	18-56	42	Samsung Galaxy S7	3 (pocket, backpack, hand)	1D-CNN-LSTM^k^	Lab	PRE	No
	Chen et al [55]	12.3 (1.0)	—	18	smartwatch “mumu”	1 (wrist)	SVM	Free-living	IC	No
	Chen et al [55]	24.9 (2.6)	—	24	smartwatch “mumu”	1 (wrist)	SVM	Free-living	IC	No
	Davoudi et al [56]	55.2 (17.8)	—	40	ActiGraph GT3X+ and Samsung smartwatch	2 (wrist)	RF	Lab	IC	No
	Doherty et al [57]	—	18-91	153	Axivity AX3	1 (wrist)	RF	Free-living	DO	No
	Ellingson et al [58]	23.9 (5.3)	—	49	ActiGraph GT3X+ and actiPAL	2 (hip, thigh)	ANN^l^ and DT^m^	Lab	IC and PRE	Yes
	Ellingson et al [59]	23.5 (4.6)	—	51	ActiGraph GT3X+	1 (hip)	RF	Lab	IC	On request
	Farrahi et al [60]	27.5 (11.2); 13.7 (3.1); 27.2 (3.3)	20-30	22; 52; 9; 8	Hookie AM20; Actigraph GT3X+; Colibri inertial measurement unit; Xsens MTx inertial measurement unit	1 (hip); 2 (hip, wrist); 1 (wrist); 2 (wrists)	ANN	Lab	DO	No
	Farrahi et al [31]	—	18-91	151	Axivity AX3	1 (wrist)	BiLSTM^n^, RF, ANN, SVM, DT, and NB^o^	Free-living	DO	No
	Freedson et al [61]	38 (12.4)	—	277	ActiGraph GT1M	1 (hip)	ANN	Lab	IC	No
	Hagenbuchner et al [29]	4.8 (0.9)	—	11	ActiGraph GT3X +	1 (hip)	ANN	Lab	PRE	No
	Hibbing et al [62]	9.4 (2.1)	—	27	ActiGraph GT3X-BT	3 (hip, both wrists)	ANN and DT	Free-living	DO	On request
	Hibbing et al [62]	10.0 (2.2)	—	54	ActiGraph GT3X+	2 (hip, wrist)	ANN and DT	Lab	IC	On request
	Li et al [63]	4.0 (0.5)	—	34	ActiGraph GT3X-BT	1 (wrist)	k-means^p^	Free-living	Hip cut points	No
	Mardini et al [64]	61.7 (17.7)	—	253	ActiGraph GT3X-BT	1 (wrist)	DT, RF, XGBoost, and LASSO^q^	Lab	IC	No
	Montoye et al [65]	22.0 (4.2)	—	40	ActiGraph GT3X+ and GENEActiv	4 (thigh, hip, both wrists)	ANN	Lab	PRE	No
	Montoye et al [66]	22.0 (4.2)	—	41	activPAL3	1 (thigh)	ANN	Lab	IC	No
	Montoye et al [67]	40.8 (19.2)	—	48	ActiGraph GT9X Link	2 (hip, wrist)	RF	Lab and free-living	DO	Yes
	Nawaratne et al [68]	45.0 (11.0)	—	119	ActiGraph GT3X +	1 (wrist)	CNN^r^	Free-living	Hip cut points	Yes
	Nnamoko et al [69]	69.3 (8.0)	—	33	GENEActiv and ActiGraph	2 (wrist, hip)	Additive regression tree	Lab	IC	No
	O'Driscoll et al [70]	44.4 (14.1); 31.9 (10.2)	—	89	ActiGraph GT3-X; SenseWear Armband	2 (wrist, upper arm)	RF, ANN, k-NN, SVM, and gradient boosting	Lab	IC	No
	Pober et al [71]	24.8 (4.2)	—	6	Actigraph MTI 7164	1 (hip)	QDA^s^ and HMM^t^	Lab	PRE	No
	Skjødt et al [72]	80.2 (3.7)	—	67	ActiGraph GT3X +, GENEActiv, and Axivity AX3	6 (both hips, both wrists, thigh, lower back)	RF	Lab	IC	Yes
	Staudenmayer et al [73]	35 (0)	21-69	48	Actigraph model 7164	1 (wrist)	ANN	Lab	IC	No
	Staudenmayer et al [74]	24.1 (0)	20-39	20	ActiGraph GT3X+	1 (wrist)	RF and DT	Lab	IC	No
	Trost et al [19]	11 (2.7)	—	100	ActiGraph GT1M	1 (hip)	ANN	Lab	IC	No
	Trost et al [19]	11 (2.7)	—	100	ActiGraph GT1M	1 (hip)	ANN	Lab	IC	No
	Trost et al [28]	4.8 (0.9)	—	11	ActiGraph GT3X +	2 (hip, wrist)	RF and SVM	Lab	DO, IC	On request
	Tsanas [75]	—	18-91	148	Axivity AX3	1 (wrist)	RF and HMM	Free-living	DO	No
	Walmsley et al [76]	—	18-91	152	Axivity AX3	1 (wrist)	RF and HMM	Free-living	DO	No
	Wang et al [33]	—	18-91	151	Axivity AX3	1 (wrist)	ViT-BiLSTM^u^, CNN-BiLSTM^v^, ViT^w^, CNN, and BiLSTM	Free-living	DO	No
	Wullems et al [77]	73.5 (6.3)	—	40	GENEActiv	2 (both thighs)	RF	Lab	IC	No
	Wullems et al [78]	70.0 (12.0)	—	20	GENEActiv	1 (thigh)	RF	Lab	DO, IC	No
	Zhou et al [79]	5.0 (0.9)	—	24	Custom inertial measurement unit sensor	1 (arm)	BiLSTM	Lab and free-living	IC	No
**Clinical conditions**										
	Bianchim et al [37]	12.0 (2.8)	—	35^CF^; 28^hy^	GENEActiv and ActiGraph	5 (both wrists, waist, both wrists)	k-NN, RF, and XGBoost	Lab	IC	No
	Cescon et al [80]	44.9 (5.0)	—	20^T1Dz^	Empatica E4 wristband	1 (wrist)	RF	Free-living	NR	No

^a^MVPA: moderate-to-vigorous physical activity.

^b^Not applicable.

^c^RF: random forest.

^d^SVM: support vector machine.

^e^DO: direct observation.

^f^PRE: predefined activity schedule.

^g^IC: indirect calorimetry.

^h^NR: not reported.

^i^k-NN: k-nearest neighbor.

^j^XGBoost: extreme gradient boosting.

^k^1D-CNN-LSTM: one directional CNN-LSTM.

^l^ANN: artificial neural network.

^m^DT: decision tree.

^n^BiLSTM: bidirectional long short-term memory.

^o^NB: naive Bayes.

^p^k-means: k-means cluster analysis.

^q^LASSO: least absolute shrinkage and selection operator.

^r^CNN: convolutional neural network.

^s^QDA: quadratic discriminant analysis.

^t^HMM: hidden Markov model.

^u^ViT-BiLSTM: vision transformer bidirectional long short-term memory.

^v^CNN-BiLSTM: convolutional neural network and bidirectional long short-term memory.

^w^ViT: vision transformer.

^x^35^CF^: 35 participants with cystic fibrosis.

^y^28^h^: 28 healthy participants in the study.

^z^20^T1D^: 20 participants with type 1 diabetes.

A total of 40 studies (2006-2025) met the inclusion criteria, with 62.5% (n=25) published between 2020 and 2025, reflecting the growing interest in AI-driven MVPA estimation.

Most studies (37/40 studies, 92.5%) targeted healthy populations, while only 5% (2/40 studies) addressed clinical cohorts, that is, cystic fibrosis [37] and type 1 diabetes [80], with one study (2.5%) did not specify the characteristics of participants [36].

Eleven studies (27.5%, 11/40) focused on children and adolescents [19,28,29,38,50-52,55,62,63,79], 40% (16/40) were on adults (18-60 years old) [34,53,55,56,58,59,61,65-68,70, 71,73,74], and 17.5% (7/40) were on old adults (60 years or older) [54,64,69,72,77,78], and 2 studies reported on the clinical conditions (ie, cystic fibrosis [37] and type 1 diabetes [80]). The remaining 7 studies (17.5%) tested their models using public datasets in adults, such as Capture-24 and Energy-24 (age range 18-91 years) [31,33,53,57,75,76] and a study with multiple datasets, including UOULU (University of Oulu), OSU (Oregon State University), the PAMAP2 Physical Activity Monitoring dataset (the UCI Machine Learning Repository), and the Daily and Sports Activities (the UCI Machine Learning Repository) [60]. Among these, Chen et al [55] covered the analyses both on children and adults; Andò et al [36], though not reporting participant ages, was contextually aligned with older adult research due to its emphasis on age-associated risks of physical inactivity among older adults and its heavy reliance on references related to older adults; Capture-24 and Energy-24 datasets [31,33,53,57,75,76] were grouped into adult, due to the age distributions: 72% of participants were younger than 53 years, with only 22.5% aged 53 years or older [82]; Farrahi et al [60], which included 4 datasets with an average participant age of about 19 years, was classified under adults.

ActiGraph (30/40, 75%) and GENEActiv (6/40, 15%) were the most common sensors using acceleration sensors to identify MVPA, with limited use of consumer wearables, for example, other brands of accelerometers (eg, Axivity AX3, activPAL; 9/40, 22.5%), inertial measurement units (5/40, 12.5%), smartwatches (2/40, 5%), smartphones (1/40, 2.5%), wristbands (1/40, 2.5%), and armbands (1/40, 2.5%).

Lab-controlled validations predominated (30/45 analyses, 66.7%; some studies had multiple analyses); 33.3% of analyses (15/45) were conducted in free-living conditions, while 4 analyses combined lab and free-living validations.

### Evolution of Feature Engineering and Model Architectures

A total of 45 analyses from 40 studies used a range of ML and DL techniques for MVPA detection.

#### Methodological Evolution

The shift from feature-driven ML to end-to-end DL reflects a broader trend toward scalability and generalizability. While traditional ML models excel in interpretability and low computational cost, their dependence on handcrafted features renders them brittle in free-living contexts. In contrast, DL architectures, though data-hungry and opaque, inherently adapt to signal variability through hierarchical abstraction, a critical advantage for real-world deployment [31].

The progression from manual feature engineering to automated DL underscores a paradigm shift toward scalable, context-aware MVPA monitoring. Table 2 synthesizes this evolution, contrasting supervised, unsupervised, and hybrid paradigms. Supervised DL models, particularly those using transfer learning, now dominate, with all studies adopting pretrained CNN or bidirectional long short-term memory (BiLSTM) to mitigate data scarcity [31,33,34,68,79]. Unsupervised approaches, such as the self-organizing map and k-means cluster analysis, remain nascent but offer potential for leveraging unlabeled free-living data [83,84].

**Table 2:** Task-specific performance comparison.

Task type and methods/ model			Key features		Performance metrics (number of studies)		References
**Classification (n=28)**							
	RF^a^ (n=13)	Handcrafted features: time/frequency features (eg, mean, SD, percentiles, lag-1 autocorrelation), ensemble of decision trees		Lab (n=7): F1-score 91.9%, accuracy 94.0% Free-living (n=4): F1-score 81.0%, accuracy 87.4% Lab and free-living (n=2): F1-score 88.1%, accuracy 93.8%		[28,36-38,50-53,64,67,72,74,80]	
	ANN^b^ (n=7)	Handcrafted features: time/frequency features (eg, spectral entropy, signal power), multilayer perceptron		Lab (n=7): F1-score 88.0%, accuracy 93.1% Free-living (n=1): F1-score 75.4%, accuracy 82.1%		[19,29,58,60,62,65,73]	
	SVM^c^ (n=4)	Kernal-based classification on RBF^d^, advanced cross-correlation metrics (xy, xz, yz)		Lab (n=1): F1-score 75.4%, accuracy 88.4% Free-living (n=3): accuracy 86.5%		[28,50,55]	
	DT^e^ (n=4)	Tree-based splits, integrate with ANN outcomes		Lab (n=3): F1-score 86.6%, accuracy 87.8% Free-living (n=1): F1-score 75.4%, accuracy 82.1%		[58,62,64,74]	
	Gradient boosting (n=3)	Gradient boosting framework, handling missing data		Lab (n=2): F1-score 91.6%		[37,54,64]	
	HMM^f^ (n=3)	Temporal sequence modeling, Viterbi smoothing		Lab (n=1): F1-score 99.8% Free-living (n=2): F1-score 73.5%, accuracy 94.0%		[71,75,76]	
	QDA^g^ (n=1)	Quadratic decision boundaries, probabilistic classification		Lab (n=1): F1-score 100%, accuracy 99.9%		[71]	
	LASSO^h^ (n=1)	L1 regularization, sparse solutions		Lab (n=1): F1-score 83.6%		[64]	
	CNN^i^ (n=1)	Automated feature extraction via convolutional filters on raw signals		Free-living (n=1): F1-score 73.4%, accuracy 96.8%		[68]	
**Estimation (n=10)**							
	RF (n=6)	Regression trees, bootstrapped subsets of ActiGraph data		Lab (n=5): F1-score 83.5%, accuracy 86.1% Free-living (n=1): F1-score 80.0%, accuracy 91.4%		[56,57,59,70,77]	
	ANN (n=2)	Nonlinear activation functions, raw signal processing		Lab (n=2): F1-score 91.1%, accuracy 85.7%		[61,66]	
	SVM (n=1)	Kernal-based regression.		Lab (n=1): F1-score 90.7%, accuracy 88.7%		[70]	
	k-NN^j^ (n=2)	Instance-based learning, Euclidean distance metrics		Lab (n=2): F1-score 96.4%, accuracy 95.8%		[37,70]	
	XGBoost^k^ (n=1)	Gradient boosting framework, handling missing data		Lab (n=1): F1-score 100%, accuracy 100%		[37]	
	Gradient boosting (n=1)	Iterative error correction, additive regression trees		Lab (n=1): F1-score 93.2%, accuracy 92.1%		[70]	
**Deep learning (n=5)**							
	Bi-LSTM^l^ (n=3)	Bidirectional temporal modeling, raw signal processing		Free-living (n=2): F1-score 73.6%, accuracy 93.6% Lab and free-living: F1-score 53.3%, accuracy 53.7%		[31,33,79]	
	CNN (n=2)	Automated feature extraction via convolutional filters on raw signals		Free-living (n=2): F1-score 71.9%, accuracy 94.4%		[33,68]	
	ViT^m^ (n=1)	Self-attention mechanisms for long-range dependencies		Free-living (n=1): F1-score 79.8%, accuracy 95.0%		[33]	
	CNN-LSTM^n^ or CNN-BiLSTM^o^ (n=2)	Hybrid architecture, integrate spatial and temporal learning		Lab (n=1): F1-score 82.1% Free-living (n=1): F1-score 91.4%, accuracy 97.7%		[33,34]	
	ViT-BiLSTM^p^ (n=1)	Vision Transformer + BiLSTM, gravity-based acceleration analysis.		Free-living (n=1): F1-score 98.4%, accuracy 99.0%		[33]	

^a^RF: random forest.

^b^ANN: artificial neural network.

^c^SVM: support vector machine.

^d^RBF: radial basis function.

^e^DT: decision tree.

^f^HMM: hidden Markov model.

^g^QDA: quadratic discriminant analysis.

^h^LASSO: least absolute shrinkage and selection operator.

^i^CNN: convolutional neural network.

^j^k-NN: k-nearest neighbor.

^k^XGBoost: extreme gradient boosting.

^l^BiLSTM: bidirectional long short-term memory.

^m^ViT: vision transformer.

^n^CNN-LSTM: convolutional neural network and bidirectional long short-term memory.

^o^CNN-BiLSTM: convolutional neural network and bidirectional long short-term memory.

^p^ViT-BiLSTM: vision transformer bidirectional long short-term memory.

#### Traditional Machine Learning

Traditional ML techniques have dominated accelerometer-based MVPA detection since 2006 [71], relying on handcrafted features derived from time- and frequency-domain analyses.

**Figure 2:**
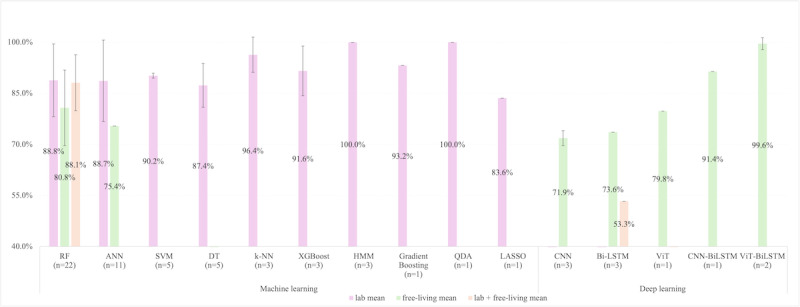
The extracted F1-score for moderate-to-vigorous physical activity from machine learning and deep learning models. Error bars, where applicable, represent SD. “n” means the number of studies using the model. ANN: artificial neural network; BiLSTM: bidirectional long short-term memory; CNN: convolutional neural network; CNN-BiLSTM: convolutional neural network and bidirectional long short-term memory; DT: decision tree; HMM: hidden Markov model; k-NN: k-nearest neighbor; LASSO: least absolute shrinkage and selection operator; QDA: quadratic discriminant analysis; RF: random forest; SVM: support vector machine; ViT: vision transformer; ViT-BiLSTM: vision transformer bidirectional long short-term memory; XGBoost: extreme gradient boosting.

Among these, RF emerged as the most prevalent algorithm (22/40 studies, 55%) [28,36-38,50-53,56,57,59,64,67,70,72, 74-78,80], achieving mean *F*_1_-scores of 86.6% and mean accuracy of 88.6%. RF’s ensemble structure, which aggregates predictions from multiple decision trees (DTs) (usually 100-1000), mitigates overfitting and enhances robustness to noise, a critical advantage in heterogeneous accelerometer datasets [67,76]. Artificial neural network (ANN) followed (11/40 studies, 27.5%), with mean *F*_1_-scores of 87.4% and mean accuracy of 89.5% [19,29,31,58,60-62,65,66,70,73]. ANN used a multi-layer perceptron with input, hidden (3-25 nodes), and output layers, nonlinear activation functions model complex feature interactions. Another often-used method was the SVM (5/40 studies, 12.5%), with an *F*_1_-score of 90.2% and accuracy of 86.5% [28,31,50,55,70]. SVM maps features to a high-dimensional space and constructs optimal hyperplanes using kernel functions (eg, radial basis function). The *F*_1_-scores of models using DT (5/40 studies, 12.5%; *F*_1_-score 87.4%) [31,58,62,64,74] underperformed k-nearest neighbor (3/40 studies, 7.5%; *F*_1_-score 96.4%) [36,37,70], extreme gradient boosting (3/40 studies, 7.5%; *F*_1_-score 91.6%) [54,64,80], hidden Markov model (3/40 studies, 7.5%; *F*_1_-score 100%) [71,75,76], Gradient Boosting (1/40 studies, 2.5%; *F*_1_-score 93.2%) [70], and quadratic discriminant analysis (1/40 studies, 2.5%; *F*_1_-score 100%) [71]. Least absolute shrinkage and selection operator achieved the lowest *F*_1_-score (83.6%) in detecting MVPA among all the ML models [64]. The details are illustrated in Figure 2.

However, performance disparities between lab-controlled and free-living environments underscored inherent limitations. RF models, for instance, exhibited a decline of 8.0% in *F*_1_-score (88.8% lab vs 80.8 free-living) and 6.6% in accuracy (90.1% lab vs 83.5% free-living), attributed to over-reliance on static features (eg, variance, spectral entropy) that fail to generalize to unstructured movement patterns [27]. Similarly, ANN experienced reduced accuracy in free-living contexts (*F*_1_-score 88.7% lab vs 75.4% free-living; accuracy 91.6% lab vs 79.4% free-living), highlighting sensitivity to signal variability introduced by nonexercise movements (eg, gesturing, device placement) [27]. The rest of the algorithms had no free-living validation.

**Figure 3:**
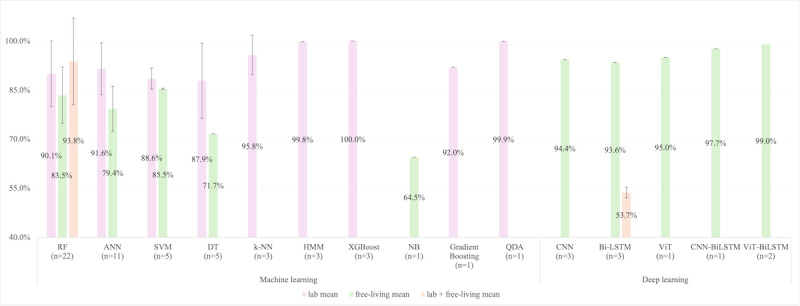
The extracted accuracy for moderate-to-vigorous physical activity from machine learning and deep learning models. Error bars, where applicable, represent SDs. “n” means the number of studies using the model. ANN: artificial neural network; BiLSTM: bidirectional long short-term memory; CNN: convolutional neural network; CNN-BiLSTM: convolutional neural network and bidirectional long short-term memory; DT: decision tree; HMM: hidden Markov model; k-NN: k-nearest neighbor; NB: naive Bayes; QDA: quadratic discriminant analysis; RF: random forest; SVM: support vector machine; ViT: vision transformer; ViT-BiLSTM: vision transformer bidirectional long short-term memory; XGBoost: extreme gradient boosting.

Figures 2 and 3 stratify *F*_1_-score and accuracy by model type, revealing that simpler algorithms like k-nearest neighbor and quadratic discriminant analysis achieved near-perfect lab performance (96.4%-100%) but performed less well in free-living validations.

#### Deep Learning

DL architectures revolutionized MVPA detection by automating hierarchical feature extraction from raw accelerometer signals, circumventing the manual feature selection bottleneck. CNN was used in 3 studies (3/40 studies, 7.5%), achieving a mean *F*_1_-score of 71.9% (shown in Figure 2) and a mean accuracy of 94.4% (shown in Figure 3) in free-living conditions [33,34,68]. Their layered structure, comprising convolutional filters (64, 128, 256, and 512 filters), pooling layers, and activation functions (such as rectified linear unit), enables granular analysis of signal dynamics.

Recurrent architectures, notably LSTM and BiLSTM, addressed temporal complexity in sustained MVPA bouts (eg, 10 min). BiLSTM, which processes sequences bidirectionally, achieves an average of *F*_1_-score 71.9% and accuracy 80.3% (shown in Figures 2 and 3) in 3 studies (of 40 studies, 7.5%) (2 in free-living settings, and 1 combining both lab and free-living settings) by modeling contextual transitions (eg, walking-to-jogging) [31,33,79]. Transformers, though less prevalent, demonstrated promise in capturing long-range dependencies through self-attention mechanisms. When hybridized with vision, vision transformer (ViT), the accuracy of detecting MVPA is 95.0% (*F*_1_-score 79.8%) in a free-living validating setting in 1 out of 40 studies (2.5%) [33].

Hybrid models (eg, convolutional neural network and bidirectional long short-term memory [CNN-BiLSTM], vision transformer bidirectional long short-term memory), in 2 out of 40 studies (5%), synergized spatial and temporal learning, achieving peak *F*_1_-score (95.5%) [34] and peak accuracy (98.4%) in free-living settings [33].

### Task-Specific Insights

#### Classification

Classification tasks in accelerometer-based MVPA research involve assigning discrete intensity classes, such as sedentary, light, moderate, or vigorous activity. Early methodologies predominantly used traditional ML algorithms, such as RF and SVM, which relied on handcrafted features like signal variance, spectral entropy, and movement counts. For instance, RF achieved robust performance in laboratory settings (*F*_1_-score mean 91.9%, Table 3) by aggregating predictions from DTs trained on bootstrapped subsets of ActiGraph data. However, these models struggled with ambiguity in free-living environments, particularly in distinguishing light-intensity activities (eg, slow walking at 2.5 METs) from MVPA (≥3 METs). SVMs with radial basis function kernels, while effective in lab-annotated running protocols (*F*_1_-score 75.4%) [28], misclassified 9.0% of slow walking (or stroll) bouts as MVPA in unstructured settings due to overlapping signal patterns [50].

**Table 3:** Taxonomy of machine learning and deep learning technologies for moderate-to-vigorous physical activity (MVPA) detection, categorized by learning paradigm.

Learning paradigm	Key features	Algorithms	Strengths	Limitations	References
Supervised	Require labeled data (activity intensity labels)	RF^a^, ANN^b^, SVM^c^, DT^d^, XGBoost^e^, HMM^f^, QDA^g^, LASSO^h^, k-NN^i^, and gradient boosting	High accuracy with sufficient labeled data Interpretable feature importance, Robust to noise and nonlinear patterns	Dependency on large, labeled datasets Overfitting risk Poor generalization to free-living environments	[28,29,33,36-38,50-60, 62,64-67,69-72,74-78,80]
Unsupervised	Work with unlabeled data, focus on clustering or feature learning	k-means, SOM^j^, and autoencoders	No need for labeled data Identifies hidden patterns in raw signals Reduces dimensionality	Limited direct applicability to MVPA classification Lower accuracy for intensity-specific tasks Interpretability challenges	[19,29,63]
Hybrid	Combine supervised and unsupervised components, integrates multiple architectures	CNN-BiLSTM^k^, ViT-BiLSTM^l^, DLEN^m^, and multitask learning frameworks	Capture spatial and temporal dependencies Improve generalizability State-of-the-art performance in free-living settings	High computational complexity Require large datasets Synchronization challenges for multisensor data	[29,31,33,34,68,79]

^a^RF: random forest.

^b^ANN: artificial neural network.

^c^SVM: support vector machine.

^d^DT: decision tree.

^e^XGBoost: extreme gradient boosting.

^f^HMM: hidden Markov model.

^g^QDA: quadratic discriminant analysis.

^h^LASSO: least absolute shrinkage and selection operator.

^i^k-NN: k-nearest neighbor.

^j^SOM: self-organizing maps.

^k^CNN-BiLSTM: convolutional neural network and bidirectional long short-term memory.

^l^ViT-BiLSTM: vision transformer bidirectional long short-term memory.

^m^DLEN: deep learning ensemble network.

DL architectures, particularly CNN, addressed these limitations by automating hierarchical feature extraction directly from raw accelerometer signals. By applying convolutional filters to sliding windows of raw data, CNN detected local biomechanical patterns (eg, stride frequency during running), achieving parity with traditional hip-based cut point methods in MVPA detection by the wrist-model [68]. Subsequent advancements, such as Transformer architectures, further improved classification accuracy by using self-attention mechanisms to model long-range dependencies, outperforming CNN by 9.5% in free-living scenarios [33].

#### Estimation

Estimation tasks focus on predicting energy expenditure metrics (eg, METs) through regression-based models to map accelerometer signals to continuous outcomes. Conventional approaches, such as linear regression-derived cut points (eg, Freedson equations), exhibited significant limitations due to oversimplified assumptions about the relationship between acceleration signals and MET values, especially for free-living activity [65,81]. ML models, such as an additive regression tree, lowered the standard error of estimation by 0.33-22.11 in lab settings using ActiGraph data [69].

DL architectures, like BiLSTM, elevated estimation accuracy by capturing temporal dependencies in accelerometer signals (eg, MET fluctuations during exercise recovery). BiLSTM achieved a mean absolute error of 0.757, with LSTM as the baseline method [79].

#### Deep Learning

DL frameworks bridge the gap between classification and estimation by unifying feature extraction and task-specific learning with end-to-end frameworks. Multitask architectures, such as AccNet24, integrate BiLSTM layers for activity intensity with fully connected layers for MET prediction, achieving 97.7% accuracy in MVPA detection in free-living settings [31].

ViTs further optimized task performance via attention mechanisms that dynamically prioritized critical signal regions. For example, ViT allocated closer attention to peak acceleration intervals during jumping, outperforming Bi-LSTM by 6.2% (*F*_1_-score) in free-living MVPA detection [33]. However, these advancements come with trade-offs; hybrid CNN-BiLSTM models require much more training time than traditional RF, limiting real-time deployment on wearables [31].

### Sensor Performance

#### Sensor Placement

**Figure 4:**
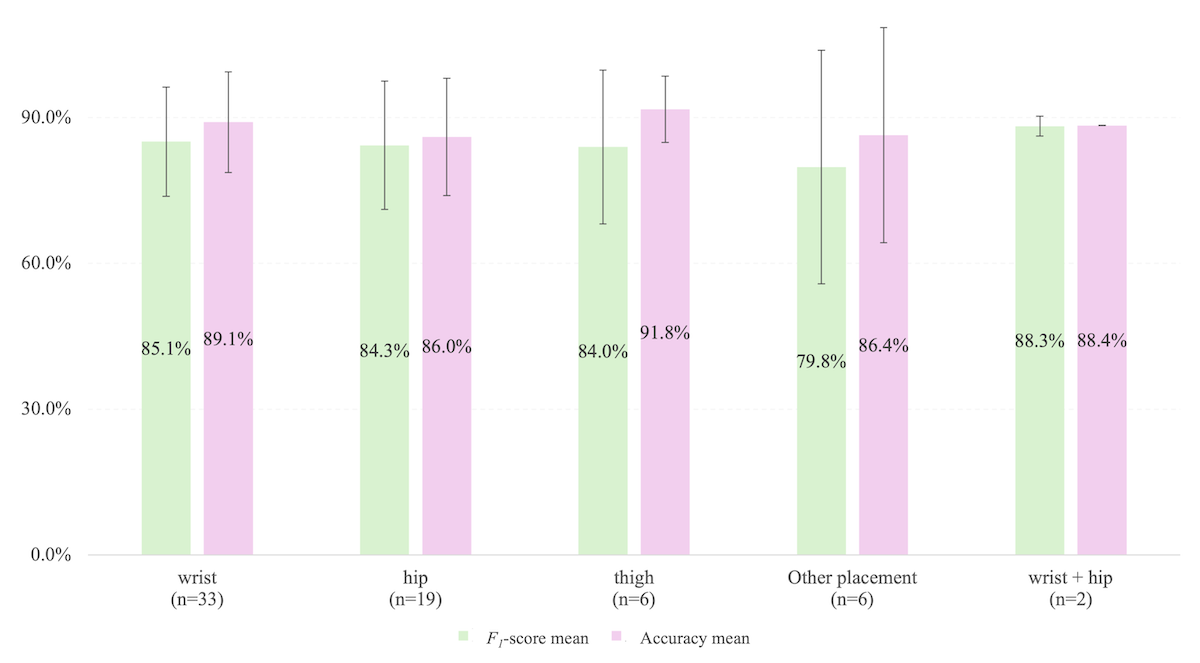
Mean value of F1-score and accuracy in relation to sensor placement across all validation settings. Error bars, where applicable, represent SDs. Other placements included chest, lower back, backpack, pocket, hand, and upper arm. “wrist + hip” means the multi-sensor configuration using both wrist and hip placements.

The efficacy of accelerometer-based MVPA estimation is significantly influenced by sensor placement. Figure 4 illustrates the averaged performance metrics (*F*_1_-scores and accuracy) across sensor placements.

Regarding sensors placement, wrist-worn devices dominated in 75% of studies (30/40 studies), followed by hip (19/40, 47.5%), thigh (6/40, 15%), chest (1/40, 2.5%), lower back (1/40, 2.5%), backpack (1/40, 2.5%), pocket (1/40, 2.5%), hand (1/40, 2.5%), upper arm (1/40, 2.5%). Multisensor configurations (eg, wrist + hip) were applied in only 5% (2/40) of studies [28,51]; only the combination of wrist and hip was applied.

Comparative analyses on MVPA revealed that wrist-, hip-, and thigh-worn sensors exhibited comparable mean performance metrics across all validation settings (mean *F*_1_-scores 85.1% vs 84.3% vs 84%, accuracy mean 89.1% vs 86% vs 91.8%) as well as in laboratory-controlled settings (*F*_1_-score mean 88.6% vs 86.6% vs 86.5%, accuracy mean 91.7% vs 89.6% vs 97.3%). However, disparities emerged in free-living environments. While wrist- and hip-worn sensors demonstrated similar *F*_1_-scores (80.3% vs 79.0%), wrist-worn devices achieved superior accuracy (86.3% vs 70.8%). This discrepancy may stem from the wrist’s ability to capture a broader range of upper-body movements associated with MVPA in unstructured environments, such as arm swings during brisk walking or lifting activities, which are less pronounced in hip-worn sensors.

Notably, multisensor configurations (eg, wrist + hip) achieved the highest performance (*F*_1_-score 88.3%, with 89.7% in lab and 86.8% in free-living; accuracy 88.4%, with 88.4% in lab but nonreports in free-living), bridging the gap between controlled and free-living settings. However, practical challenges, including increased participant burden due to multiple devices and synchronization complexities between heterogeneous sensors, limit their widespread adoption.

#### Sensor Type and Performance Heterogeneity

**Figure 5:**
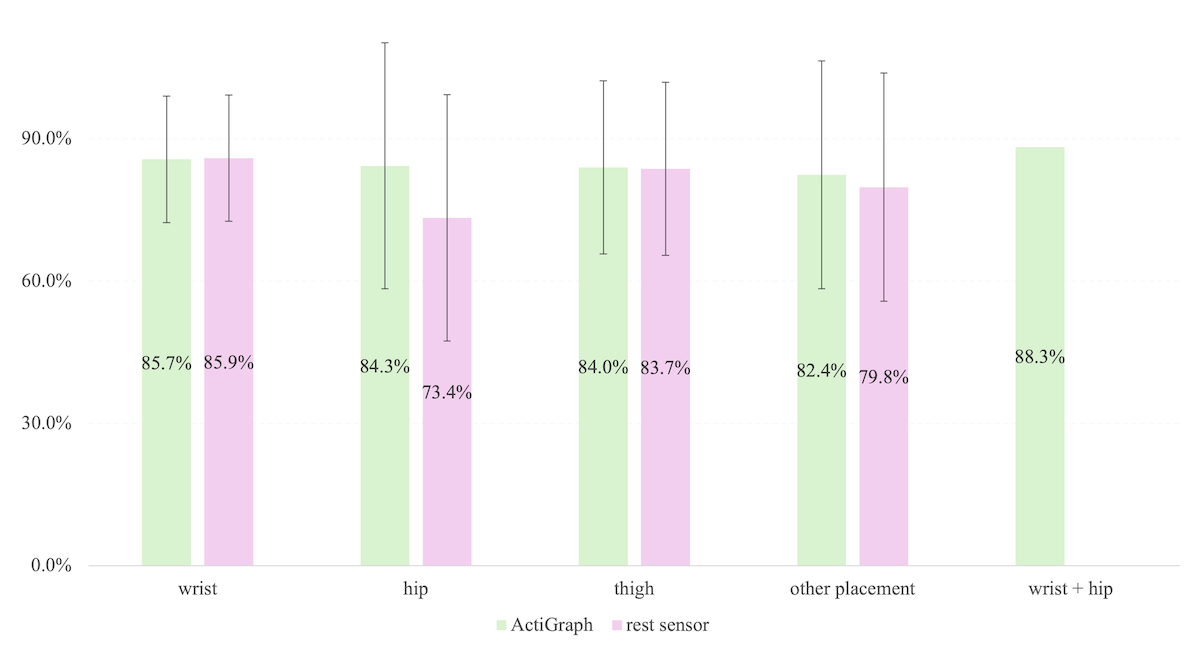
Mean value of F1-score in relation to sensor type and placement across all validation settings. Error bars, where applicable, represent SDs.

Device specifications and sensor type further influenced model generalizability. Figure 5 shows the *F*_1_-scores in relation to sensor type (ActiGraph and other types) and sensor placement. While models trained on ActiGraph data achieved a higher mean *F*_1_-score (84.9% vs 83.1%), models using consumer-grade wearables surprisingly achieved a higher mean accuracy (91.8% vs 87.8%). This disparity may arise from the inherent class imbalance in free-living data, where MVPA represents a minority of activities. Accuracy can be inflated by correctly classifying the predominant sedentary and light activities, whereas the *F*_1_-score provides a more balanced measure of performance specifically for the MVPA class. The higher *F*_1_-score associated with ActiGraph models suggests they may be more adept at correctly identifying true MVPA bouts, which is critical for public health monitoring.

### Validation Practices

#### Ground Truth Methodologies and Their Implications

Validation of ML and DL models for MVPA detection relies heavily on the specification of ground truth, with IC and DO predominating. IC (21/40 studies, 52.5%), considered the gold standard for energy expenditure measurement, provides MET values through oxygen consumption analysis, enabling precise alignment of accelerometer signals with intensity thresholds (eg, ≥3 METs) [19,28,37,53-56,58,59,61,62,64,66,69,70, 72-74,77-79]. However, its laboratory-bound nature limits ecological validity, as structured protocols often fail to replicate free-living movement variability.

In contrast, DO (16/45 analyses, 35.6%) offers real-world applicability by annotating activities in a naturalistic setting but introduces subjectivity, particularly in distinguishing borderline intensities with 2 stages. At the first stage, participant movements were categorized into activity type (eg, sedentary, standing utilitarian tasks, walking, and running) using recordings [28,38,50,51,60,62,65,67,78] and time-stamped images from wearable cameras (eg, combining the usage of a diary in Capture-24) [31,33,53,57,75,76]. At the second stage, physical intensity was coded using references, mainly the Compendium of Physical Activities (sedentary, light, moderate, and vigorous) and Children’s Activity Rating Scale (5 categories from stationary/motionless to fast translocation).

Additionally, reliance on hip-reference cut points as proxies for ground truth (2/45 analyses, 4.4%) perpetuates circular validation, wherein models trained on threshold-based labels inherit the biases of traditional regression methods [63,68]. A total of 15.6% (7/45) of analyses used a predefined activity schedule in the validation process to define the ground truth [29,36,52,58,71]. Only 11.1% (5/45) of analyses used combined ground truth approaches (eg, IC + DO) [28,53,78], despite evidence that combined methods improve *F*_1_-score by 8.3%-27.7% in free-living compared with IC or DO [53].

**Figure 6:**
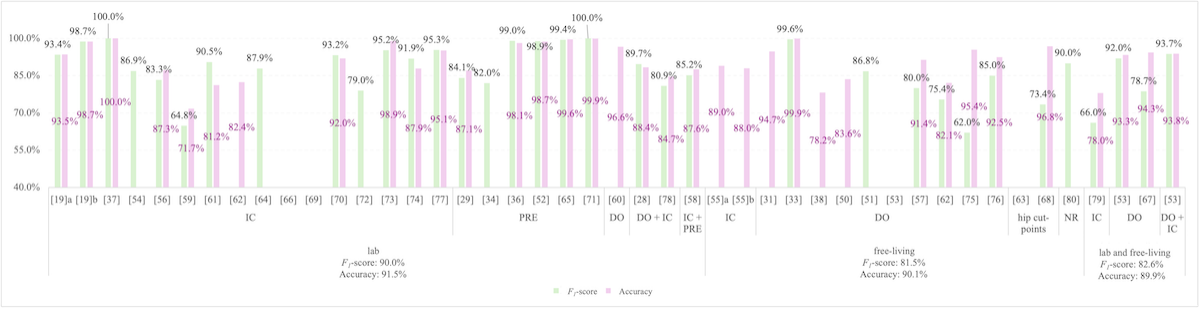
F1-score and accuracy metrics for moderate-to-vigorous physical activity (MVPA) classification across studies, stratified by ground truth methods and validation settings. [19]a represents the MVPA classification conducted based on the 10s and 60s window lengths, while [19]b only on the 60s window length. [55]a represents the MVPA classification conducted among the children and adolescents with a mean age of 12.3 (SD 1.0) years, while [55]b represents the MVPA classification conducted among adults aged 24.9 (SD 2.6) years. The mean value of F1-score and accuracy in each group (lab, free-living, and lab and free-living) was shown underneath their names [19,28,29,31,33,34,36-38,50-80]. DO: direct observation; hip: hip reference cut-points; IC: indirect calorimetry; NR: not reported; PRE: predefine activity schedule.

Figure 6 illustrates the distribution of ground truth methods across studies, stratified by validation setting. Lab-based studies disproportionately favored IC (19/26 analyses, 73%), while free-living validations leaned on DO (10/15 analyses, 66.7%), with lab validations outperforming free-living (*F*_1_-score mean difference: 8.5%, accuracy mean difference: 1.4%).

#### Cross-Validation Protocols and Performance Metrics

The trained models typically undergo rigorous evaluation during the model evaluation and validation phase to verify their generalizability and practical applicability. This process is essential to systematically assess classification accuracy across activity intensities and validate reliability under diverse user scenarios. To mitigate overfitting and ensure model robustness, *k*-fold cross-validation (11/45 analyses, 24.4%) was most commonly implemented. It partitions the dataset into *k* equally sized subsets, iteratively designating one subset as the validation set and the remaining *k*-1 subsets for training. The process is repeated *k* times to ensure all data points contribute to both training and validation. Common configurations include 10-fold and *5*-fold cross-validation, which enhance model generalizability by reducing sensitivity to specific training instances. By systematically evaluating performance across varied data partitions, this method strengthens the activity intensity classification system’s reliability and mitigates overfitting, a phenomenon where models memorize training data artifacts rather than learning generalizable patterns.

Leave-one-out cross-validation, which iteratively holds out each individual data point as a test set to evaluate model performance, was also used in 13.3% of analyses (6/45 analyses). Leave-one-subject-out cross-validation, an extension of leave-one-out cross-validation designed for datasets with multiple subjects, iteratively holds out all data from one subject as the test set while training on the remaining participants. This method, used in 24.4% of analyses (11/45), was critical for assessing interindividual generalizability. Its variant, leave-10-subject-out cross-validation, appeared less frequently (1/45 analysis). In contrast, nested cross-validation, which separates hyperparameter tuning from final performance evaluation to prevent data leakage, was sparingly adopted (3/45 analyses, 6.7%) in studies.

Model efficacy was quantified using precision, recall, accuracy, and *F*_1_-score, with metrics calculated iteratively to ensure objective assessment. *F*_1_-score (33/45 analyses, 73.3%) and accuracy (35/45 analyses, 77.8%) were emphasized in this review, though their interpretation varied widely. Notably, studies reporting accuracy exceeding 90.0% often excluded transitional activities (eg, sit-to-stand) [19,37,65,70] or used imbalanced datasets [28,71,80], potentially inflating scores. Conversely, *F*_1_-scores below 75.0% typically correlated with free-living validations, where non-MVPA movements confounded detection [62,68,75].

In the studies not reporting the metrics of MVPA detection, but including a confusion matrix, we calculated *F*_1_-scores and accuracy values from the confusion matrix [28,29,33,36,52,53,57,58,61,62,67,68,70,71,73-78]. Two studies reported the *F*_1_-scores of moderate physical activity (MPA) and vigorous physical activity (VPA) separately without a confusion matrix, so they averaged MPA and VPA to get MVPA [72,79].

Four studies omitted both *F*_1_-score and accuracy [53,63,66,69]. For instance, Li et al [63] reported only the overall accuracy of physical activity intensity classification, while Montoye et al [66] quantified MVPA error (+1.8 min) relative to IC. Ahmadi et al [53] only provided sensitivity (MPA: 80.0%, VPA: 90.0%) and precision (MPA≈75.0%, VPA≈99.0%), and Nnamoko et al [69] reported only the standard error for estimation of personalized cut points.

### Algorithmic Bias

The performance of machine and DL models for MVPA detection is inherently tied to the physiological characteristics of the training populations. These have been mostly young, healthy adults. Persistent algorithmic bias induced by using this group can undermine the generalizability of models across the older adult and clinical cohorts.

**Figure 7:**
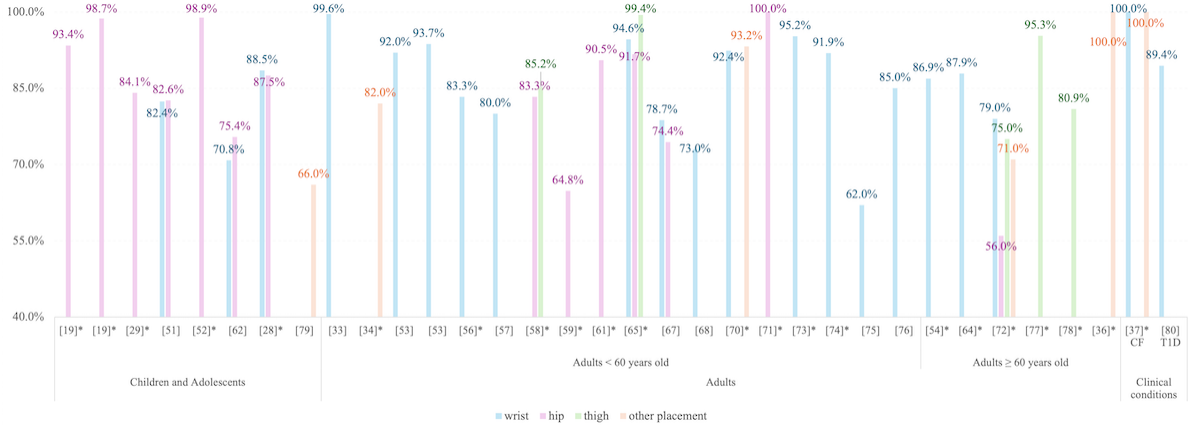
F1-scores for moderate-to-vigorous physical activity detection across age groups and sensor placements. Brackets (“[]”) represent the reference numbers; asterisks (“*”) indicate lab-validated results [19,28,29,33,34,36,37,51-54,56-59,61,62,64,65,67,68,70-80]. CF: cystic fibrosis; T1D: type 1 diabetes.

**Figure 8:**
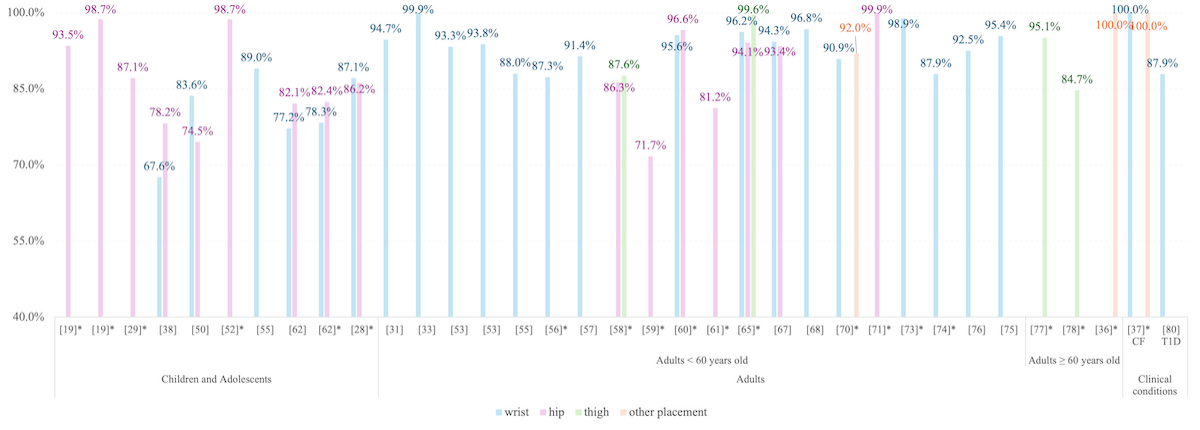
Accuracy metrics for moderate-to-vigorous physical activity detection across age groups and sensor placements. Brackets (“[]”) represent the reference numbers; asterisks (“*”) indicate lab-validated results [19,28,29,31,33,36-38,50,52,53,55-62,65,67,68,70,71,73-78,80]. CF: cystic fibrosis; T1D: type 1 diabetes.

Figures 7 and 8 stratified MVPA detection performance (*F*_1_-scores and accuracy) by age group (children and adolescents, adults younger than 60 years, adults aged 60 years or older, and clinical populations) and sensor placement (wrist, hip, thigh, other). Among children and adolescents (11/40 studies, 27.5%), both *F*_1_-scores (53.5%-98.9%) and accuracy (52.1%-98.7%) varied widely [19,28,29,38,50-52,55,62,63,79]. Adults under 60 years (20/40 studies, 50%) exhibited consistently high performance (*F*_1_-score mean 85.8%, accuracy mean 91.7%) [31,33,34,53,55-59,61,65-68,70,71,73-76], while older adults (60 years or older) in 15% of studies (6 out of 40 studies) showed relatively reduced score (*F*_1_-score mean 72.3%, accuracy mean 89.9%) [54,64,69,72,77,78]. Clinical populations (2/40 studies, 5%), on the other hand, achieved near-perfect scores (*F*_1_-score 97.6%-100%, accuracy 87.9%-100%), though limited studies (n=2, one in mild cystic fibrosis, and one in type 1 diabetes) necessitate cautious interpretation [37,80].

### Reproducibility and Transparency Gaps

Notwithstanding the performance advancement reported, 57.5% of the studies (23/40 studies) failed to disclose code or datasets, and 60% (3/5 studies) of DL studies lacked hyperparameter specifications (eg, learning rates, batch sizes) [34,68,79]. This “black box” methodological opacity mirrors the reproducibility crisis in traditional cut point research, where proprietary algorithms replace opaque thresholds, compromising interpretability. Only 20.0% of studies (8/40) adhered to open science practices by publicly sharing the code of models [38,51,52,58,67,68,72,76], with one study providing only sample code availability [67].

The absence of standardized reporting frameworks exacerbates methodological inconsistencies. For example, window lengths for signal segmentation ranged from 1s to 60s, complicating cross-study comparisons (details shown in Table 1). While 5 studies evaluated multiple window lengths [19,29,33,51,56], optimal performance diverged across populations: 15s or 16s were preferred for adults [33,56] and preschoolers [51], whereas 60s windows were superior to 10s and 30s among preschoolers [29]. Notably, Trost et al [19] found no difference between 10s and 60s among children (mean 11, SD 2.7 years). Similarly, MET thresholds for MVPA classification varied, such as 2.8 METs [38], 3 METs [34,37,59,60,65-67,70,71,74,76,77,79], and 3.9 METs [19,29]. This variability introduces heterogeneity in intensity categorization, undermining cross-study generalizability.

On the positive side, the proliferation of public datasets (accounting to 25%, 10/40 studies), including Capture-24 [31,33,53,57,75,76], Energy-24 [57], UOULU (University of Oulu) [60], OSU (Oregon State University) [60], the PAMAP2 Physical Activity Monitoring dataset (University of California, Irvine, UCI) [60], and the Daily and Sports Activities (the UCI Machine Learning Repository) [60] has partially mitigated by enabling benchmarking and reducing data dependency.

## Discussion

### Principal Findings

This systematic scoping review synthesizes advancements in ML and DL techniques for estimating and predicting MVPA from accelerometer data. Traditional ML models (eg, RF, ANN) demonstrated robust lab-based accuracy (*F*_1_-score mean 83.6%-100%) while real-world performance declined by 8.0%-13.3% due to environmental noise and device heterogeneity. DL architectures (eg, CNN, Transformer) achieved superior performance by leveraging raw signal dynamics (*F*_1_-score mean 73.6%-98.4%) in free-living settings, especially with hybrid models (CNN-BILSTM, ViT-LSTM). Wrist-worn devices were most often tested (30/40, 75.0% of studies) and performed comparably to hip/thigh placements in a lab setting (*F*_1_-score mean 84.0%-84.3%, accuracy mean 86.0%-91.8%). Multiaccelerometer configurations (eg, hip + wrist) achieved the best performance (accuracy mean 88.4%) but face practical limitations. Algorithmic bias was seen to disfavor older adult participants, but not clinical populations. However, only a few studies have tested patients, limited to cystic fibrosis and type 1 diabetes.

### Methodological Advancements and Challenges

#### From Feature Engineering to End-to-End Learning

The evolution of MVPA detection methodologies reveals a clear paradigm shift from manual feature engineering to automated DL. Historically, ML models relied on handcrafted features (eg, spectral entropy, variance) derived from time-and frequency-domain analyses. In contrast, DL architectures, such as CNN and Transformers, automate hierarchical feature extraction from raw accelerometer signals, capturing biomechanical nuances (eg, stride variability during running) through convolutional filters and attention mechanisms [33,68]. For instance, hybrid models like CNN-BiLSTM synergized spatial and temporal learning, achieving state-of-the-art accuracy (*F*_1_-score 98.4%-99.6%, accuracy 97.7%-99.0%) in free-living settings [33]. The effectiveness of this architecture is further corroborated by its successful application in related biomechanical modeling tasks, such as predicting ligament fatigue failure risk from complex signal data, highlighting its robust capability to capture critical spatiotemporal patterns [85]. Nevertheless, DL’s computational intensity and reliance on high-resolution data (≥100Hz) limited deployment on resource-constrained wearables [33]. Furthermore, while DL reduced manual feature engineering burden, nearly 60.0% of models remained inaccessible due to unshared code, perpetuating reproducibility challenges (section “Reproducibility and Transparency Gaps”).

Three key advancements define this evolution: (1) Static to dynamic features, unlike the fixed features of traditional ML, DL architectures dynamically extract nuanced biomechanical patterns from raw signals (section “Traditional Machine Learning” and “Deep Learning”) [31,33,34,68,79]. (2) Early studies treated classification and estimation as separate tasks, but modern frameworks like AccNet24 unify these through shared neural pathways, improving efficiency (section “Task-Specific Insights”) [33]. (3) Self-supervised learning, pretraining on unlabeled data, reduced annotation costs while maintaining high performance, addressing scarcity of free-living settings (section “Methodological Evolution and Comparative Insights”) [33,68].

#### Lab-to-Real-World Performance Comparison

Although lab-validated models achieved high performance (eg, 87.9%-100% accuracy across ML techniques, section “Evolution of Feature Engineering and Model Architectures”), free-living performance experienced unstructured movement patterns and environment noise. For example, RF accuracy dropped from 90.1% (lab) to 83.5% (free-living), while wrist-based models exhibited superior adaptability to upper-body movements (eg, arm swings) in unstructured settings (section “Sensor Placement”). Notably, only 42.2% of studies validated models in real-world environments (most after 2020), highlighting a critical translational gap.

Two key insights emerge from a lab-to-real-world comparison. (1) There is a 3.1%-16.2% accuracy decline when using ML techniques (section “Evolution of Feature Engineering and Model Architectures”). Context-aware architecture, DL architectures, such as transformers, partially mitigated performance declines by leveraging context-aware attention to movement sequences (eg, detecting walking interruptions), achieving accuracy of 95.0% in free-living scenarios [33]. (2) There is an algorithmic bias across age groups that hinders real-world deployment (section “Algorithmic Bias”).

#### Validation and Reproducibility

A key challenge lies in inconsistent validation protocols. While IC provided precise MET-based thresholds, its lab-bound nature limited ecological validity [58]. Conversely, DO offered real-world applicability but introduced subjectivity in intensity classification [86,87]. Moreover, disparities in metrics reporting (eg, exclusion of transitional activities) [19,37,65,70] and variable parameters (eg, MET threshold: 2.8-3.9, window lengths 1-60s) hindered cross-study comparability (section “Reproducibility and Transparency Gaps”). Compounding these issues, 42.5% of studies adhered to open science practices, perpetuating a “new cut-point conundrum” akin to proprietary regression thresholds.

Our synthesis reveals a vicious cycle underpinning the translational challenges in AI-driven MVPA monitoring. The foundational issue is the lack of standardized validation protocols. Inconsistent MET thresholds and variable data window lengths mean that models are trained and evaluated on fundamentally different definitions of MVPA. This directly contributes to the lab-to-real-world performance gap, as a model calibrated with one protocol fails to generalize to data collected under another. Furthermore, this inconsistency, when combined with the prevalent lack of code sharing, makes it impossible to audit, replicate, or fairly compare models. Consequently, this opacity hinders the identification and correction of algorithmic bias against underrepresented populations, as the root cause of poor performance, a flawed model versus an incompatible validation method, cannot be discerned. Thus, these challenges are not isolated but are synergistic barriers that collectively impede the development of truly generalizable and equitable models.

#### Sensor Performance and Device Bias

Device placement and type emerged as critical determinants of model performance, as evidenced in the section “Sensor Performance.” For instance, while ActiGraph-trained models achieved high lab accuracy (*F*_1_-score 79.9%, accuracy 90.5%), they underperformed on consumer wearables (eg, Samsung smartwatch, *F*_1_-score mean difference 3.2%) due to differences in sensor calibration and sampling rates (Table 1 and Figure 5) [56]. Additionally, interdevice variability across brands (eg, Axivity vs GENEActiv) exacerbated performance inconsistencies, particularly in free-living settings. Notably, optimal sensor placement (eg, wrist vs hip) influenced adaptability to movement patterns, with wrist-worn devices showing superior capture of upper-body dynamics (eg, arm swings) but struggling with lower-body activities [40,88]. These findings highlight the need for device-agnostic training pipelines to mitigate performance variability across brands and placements.

### Translational Opportunities and Challenges

#### Public Health and Clinical Integration

Wrist-worn devices demonstrated comparable accuracy to hip/thigh placements in lab settings (*F*_1_-score 84.0%-84.3%, accuracy 86.0%-91.8%) and superior adaptability to free-living upper-body movements (Figure 4), supporting their feasibility for scalable monitoring. However, ActiGraph’s dominance (n=30, 75.0% of studies) and limited validation on consumer wearables (eg, smartwatches) hinder real-world applicability. Clinically, models achieved high accuracy in controlled settings for cystic fibrosis and type 1 diabetes, but small sample sizes and structured protocols limit ecological validity [37,80]. Expanding validation studies to more diverse clinical populations (eg, mobility impairments) is critical.

#### Age and Population Disparities

Results revealed systemic biases across the age range (section “Algorithmic Bias”). For instance, models trained on adults misclassified MVPA in children (*F*_1_-score mean difference: –5.7%) due to developmental differences in stride length and metabolic variability [19]. Studies involving preschoolers reported accuracy fluctuations between 53.7% and 88.4%, reflecting challenges in modeling erratic movement patterns typical of young children [28,29,38,50,52,63,79]. Conversely, older adults (60 years or older) exhibited reduced accuracy (*F*_1_-score mean 77.9%) due to slower gait speeds, postural instability, and comorbidities that alter movement signatures [54,64,69,72,77,78]. Wrist-based model, for example, underestimated MVPA in this cohort by 6.0%-16.6% compared with thigh-worn sensors, highlighting the need for age-specific calibration [78].

### Emerging Innovations

Hybrid DL models have emerged as a powerful approach. For instance, integrating LSTM with CNN (CNN-LSTM) or ViTs (vision transformer bidirectional long short-term memory) enables the capture of spatial-temporal patterns in accelerometry data [33,34]. Building on this, BiLSTM layers further enhance temporal dependency modeling by analyzing sequences in both forward and backward directions [31].

In parallel, image-based feature extraction methods, such as converting raw accelerometer signals into Gramian angular field images, have improved feature learning by transforming time-series data into visual representations [33]. Additionally, multisensor fusion strategies—combining data from hip, wrist, and thigh placements—address variability in sensor positioning, boosting model robustness [28,34]. Furthermore, transfer learning leverages pretrained architectures like ResNet101, adapting them for accelerometer classification tasks [31].

Another key innovation lies in advanced feature engineering. Autonomous feature extraction via CNN reduces reliance on handcrafted features [68], while time-frequency domain fusion (eg, spectral power) enhances activity discrimination [52]. Notably, real-time and edge computing advancements explore lightweight models through pruning and quantization, enabling deployment on wearable devices [33].

However, significant challenges remain. First, models trained in controlled lab settings often generalize poorly to free-living environments due to uncontrolled variability [52]. Moreover, short and heterogeneous activity bouts, common in populations like preschoolers, result in mixed-activity windows that complicate classification [29]. Another critical challenge is sensor placement variability, as signal patterns differ across body positions [37]. Compounding this, class imbalance from overrepresented sedentary/light activities skews model performance [75]. Additionally, computational complexity limits real-time use, as seen in resource-heavy models like AccNet24 [31]. Finally, distinguishing biomechanically similar activities (eg, climbing vs walking) remains problematic [29].

### Future Directions

To address existing gaps, 4 interconnected priorities emerge. First, resolving inconsistencies in ground truth methods, such as variable MET thresholds (2.8-3.9 METs) and window lengths (1-60s), is critical. This requires standardized validation frameworks, including consensus guidelines and open datasets (eg, Capture-24), to harmonize protocols and reduce discrepancies in intensity classification [64].

Second, prioritizing free-living validation is essential to bridge the lab-to-real-world performance gap. For instance, RF models exhibit accuracy declines from 90.1% in lab settings to 83.5% in free-living environments. Concurrently, diversifying training data to include underrepresented groups, such as the older adult, pediatric, and clinical populations, will improve generalizability and mitigate age-related biases [62,72].

Third, advancing algorithmic fairness through regulatory frameworks and bias audits is imperative. This includes expanding datasets to encompass more and diverse clinical cohorts while addressing disparities in model performance across the age range. Additionally, mandating open science practices, such as code/data sharing and hyperparameter transparency, will enhance reproducibility and resolve the “new cut-point conundrum” plaguing activity intensity thresholds.

Finally, optimizing DL architectures, such as quantized models or hybrid CNN-BiLSTM frameworks, for low-power wearables will enable real-world deployment while maintaining computational efficiency [31,33].

Looking ahead, these priorities align with broader calls for standardization and interpretability. For example, improving the “black-box” nature of DL models [68] and harmonizing evaluation metrics will foster clinical trust. Moreover, lightweight, edge-compatible architectures and multimodal data integration represent promising pathways to overcome current limitations in real-world MVPA monitoring.

### Limitations and Methodological Considerations

The strengths of this review include the rigorous adherence to PRISMA-ScR guidelines, a comprehensive search strategy across 3 electronic databases (PubMed, IEEE Xplore, Web of Science, and others via manual citation tracking), and systematic screening of 1938 records. The methodology prioritized transparency through dual-reviewer full-text screening to resolve discrepancies and consultation to ensure methodological rigor. By focusing on peer-reviewed studies, we aimed to synthesize evidence grounded in empirical validation, thereby minimizing inclusion of speculative or opinion-based articles.

However, several limitations warrant consideration. First, the exclusion of gray literature (eg, unpublished trials, industry reports, or conference proceedings) may have omitted insights from ongoing or unsuccessful implementation efforts, particularly those led by technology developers or health care providers. This introduces potential publication bias, as negative results or pragmatic challenges in real-world deployment are often underrepresented in peer-reviewed journals. Second, our decision to exclude non–peer-reviewed studies and prioritize articles reporting empirical implementation in clinical or free-living settings risks overlooking formative research, such as feasibility studies or pilot trials, which could offer valuable lessons for scalable AI integration.

A further limitation arises from our emphasis on the highest reported performance metrics (eg, *F*_1_-scores, accuracy) across studies. While this approach highlights peak algorithmic capabilities, it may overestimate real-world applicability, as optimal configurations (eg, 15-second windows for adults, multisensor placements) often lack generalizability to diverse populations or unstructured environments. For instance, models achieving 99.0% accuracy in lab settings may exhibit significant performance degradation in free-living contexts due to uncontrolled variables like device heterogeneity or nonexercise movements.

Methodologically, while the Arksey and O’Malley framework does not mandate quality appraisal, the inclusion of studies with heterogeneous validation protocols (eg, variable MET thresholds, ground-truth methodologies) complicates cross-study comparisons. Future reviews could strengthen synthesis by incorporating quality assessment tools to evaluate bias risk and methodological consistency. Last, the predominance of studies using young, healthy cohorts limits insights into algorithmic fairness and generalizability for older adult or clinical populations, underscoring the need for more inclusive training datasets.

These considerations do not diminish the review’s contributions but highlight critical gaps, such as reproducibility challenges and translational biases, that must be addressed to advance equitable, real-world deployment of AI-driven MVPA monitoring tools.

### Conclusions

This systematic scoping review highlights that ML and DL have significantly advanced in the detection of MVPA by using accelerometer data, yet persistent gaps in generalizability and transparency hinder real-world impact. To bridge the lab-to-real-world divide, collaborative efforts across public health and computer science must prioritize reproducibility, inclusive design, and robust validation. By addressing these challenges, AI-driven tools can fulfill their potential as scalable, equitable solutions for advancing global physical activity research and intervention.

## Appendix

Multimedia Appendix 1 Search strategy.

Multimedia Appendix 2 The full summary of included studies (N=40 studies, ranked by health condition and alphabets of author names).

Multimedia Appendix 3 PRISMA-ScR checklist.

## Abbreviations

AI: artificial intelligence
ANN: artificial neural network
BiLSTM: bidirectional long short-term memory
CNN: convolutional neural network
CNN-BiLSTM: convolutional neural network and bidirectional long short-term memory
CNN-LSTM: convolutional neural network and long short-term memory
DL: deep learning
DO: direct observation
DT: decision tree
IC: indirect calorimetry
LSTM: long short-term memory network
MET: metabolic equivalent
ML: machine learning
MPA: moderate physical activity
MVPA: moderate-to-vigorous physical activity
OSU: Oregon State University
PRISMA: Preferred Reporting Items for Systematic Reviews and Meta-Analyses
PRISMA-ScR: The Preferred Reporting Items for Systematic Reviews and Meta-Analyses Extension for Scoping Reviews
RF: random forest
RQ: research questions
SVM: support vector machine
UOULU: University of Oulu
ViT: vision transformer
VPA: vigorous physical activity
