# Supplementary material for: Machine and Deep Learning for Detection of Moderate-to-Vigorous Physical Activity From Accelerometer Data: Systematic Scoping Review
Source: Interact J Med Res. 2026 Jan 8;15:e76601. doi: 10.2196/76601 (PMC12828321; doi:10.2196/76601)
Supplement: Multimedia Appendix 1 [file ijmr_v15i1e76601_app1.docx]

**Multimedia Appendix 1. Search strategy.**

The search conducted on April 7, 2025

**Searching strategy**

| **Subterm** | **Descriptors** |
| --- | --- |
| **Artificial Intelligence** | “artificial intelligence” OR “machine learning” OR “deep learning” OR “neural network*” OR “supervised learning” OR “unsupervised learning” OR “reinforcement learning” OR “computer vision” OR “pattern recognition” OR “random forest” OR “support vector machine*” OR “long short-term memory” OR “decision tree*" OR “gradient boosting” |
| **Accelerometer Data** | Acceleromet* OR “activit* monitor*” OR “wearable device*” OR “smart device*” OR “wearable technology” OR smartwatch* OR smartphone OR “smart phone” OR ActiGraph OR “inertial measurement unit*” OR IMU |
| **MVPA** | “moderate to vigorous physical activit*” OR MVPA OR “moderate-to-vigorous physical activit*” OR “moderate physical activit*” OR “moderate intensit*” OR MPA or “vigorous physical activit*” OR “vigorous intensit*” OR “VPA” OR “intensive physical activit*” OR “intensive intensit*” OR run OR “stair climb*” OR “energy expend*” OR “physical activity intensit*” OR “physical intensit*” |
| Overall search term structure | “**Artificial Intelligence**” AND “**Accelerometer Data**” AND “**MVPA**” |

**PubMed**

| **Subterm** | **Descriptors** | **Number of studies reached** |
| --- | --- | --- |
| **Artificial Intelligence** | (artifical intelligence[MeSH Terms]) OR ("artificial intelligence"[Title/Abstract] OR "machine learning"[Title/Abstract] OR "deep learning"[Title/Abstract] OR "neural network*"[Title/Abstract] OR "supervised learning"[Title/Abstract] OR "unsupervised learning"[Title/Abstract] OR "reinforcement learning"[Title/Abstract] OR "computer vision"[Title/Abstract] OR "pattern recognition"[Title/Abstract] OR "random forest"[Title/Abstract] OR "support vector machine* "[Title/Abstract] OR "long short-term memory"[Title/Abstract] OR "decision tree*"[Title/Abstract] OR "gradient boosting"[Title/Abstract]) | 434,222 |
| **Accelerometer Data** | (Acceleromet*[Title/Abstract] OR "activit* monitor*"[Title/Abstract] OR "wearable device*"[Title/Abstract] OR "smart device*"[Title/Abstract] OR "wearable technology"[Title/Abstract] OR smartwatch*[Title/Abstract] OR smartphone[Title/Abstract] OR "smart phone"[Title/Abstract] OR ActiGraph[Title/Abstract] OR “inertial measurement unit*”[Title/Abstract] OR IMU[Title/Abstract]) | 69,341 |
| **MVPA** | ("moderate to vigorous physical activit*"[Title/Abstract] OR MVPA[Title/Abstract] OR "moderate-to-vigorous physical activit*"[Title/Abstract] OR "moderate physical activit*"[Title/Abstract] OR "moderate intensit*"[Title/Abstract] OR MPA[Title/Abstract] OR "vigorous physical activit*"[Title/Abstract] OR "vigorous intensit*"[Title/Abstract] OR "VPA"[Title/Abstract] OR "intensive physical activit*"[Title/Abstract] OR "intensive intensit*"[Title/Abstract] OR run[Title/Abstract] OR "stair climb*"[Title/Abstract] OR "energy expend*"[Title/Abstract] OR "physical activity intensit*"[Title/Abstract] OR "physical intensit*"[Title/Abstract]) | 232,900 |
| Overall search term structure | (“**Artificial Intelligence**” AND “**Accelerometer Data**” AND “**MVPA**”) | 209 |

Additional filters were added: article language (choose only “English”), species (choose “humans”)

**Web of science**

Search in “All Databases”

Editions: “All”

| **Subterm** | **Descriptors** | **Number of studies reached** |
| --- | --- | --- |
| **Artificial Intelligence** | TS=(“artificial intelligence” OR “machine learning” OR “deep learning” OR “neural network*” OR “supervised learning” OR “unsupervised learning” OR “reinforcement learning” OR “computer vision” OR “pattern recognition” OR “random forest” OR “support vector machine*” OR “long short-term memory” OR “decision tree*" OR “gradient boosting”) | 1,944,659 |
| **Accelerometer Data** | TS=(Acceleromet* OR “activit* monitor*” OR “wearable device*” OR “smart device*” OR “wearable technology” OR smartwatch* OR smartphone OR “smart phone” OR ActiGraph OR “inertial measurement unit*” OR IMU) | 187,281 |
| **MVPA** | TS=(“moderate to vigorous physical activit*” OR MVPA OR “moderate-to-vigorous physical activit*” OR “moderate physical activit*” OR “moderate intensit*” OR MPA or “vigorous physical activit*” OR “vigorous intensit*” OR “VPA” OR “intensive physical activit*” OR “intensive intensit*” OR run OR “stair climb*” OR “energy expend*” OR “physical activity intensit*” OR “physical intensit*”) | 1,319,227 |
| Overall search term structure | (“**Artificial Intelligence**” AND “**Accelerometer Data**” AND “**MVPA**”) | 1542 |

Additional filters were added: Language (choose only “English”), other (choose “exclude preprints”), “Document Types” (exclude “review”, “Dissertation Thesis”, “Abstract”)

**IEEE Xplore**

Search in “All Databases”

| **Subterm** | **Descriptors** | **Number of studies reached** |
| --- | --- | --- |
| **Artificial Intelligence** | “artificial intelligence” OR “machine learning” OR “deep learning” | 719,828 |
| **Accelerometer Data** | Acceleromet* | 35,389 |
| **MVPA** | “moderate-to-vigorous physical activit*” OR “moderate physical activit*” OR “vigorous physical activit*” OR “energy expend*” OR “activity intensit*” OR “intensive intensit*” | 99,830 |
| Overall search term structure | (“**Artificial Intelligence**” AND “**Accelerometer Data**” AND “**MVPA**”) | 187 |
