# Supplementary material for: Machine and Deep Learning for Detection of Moderate-to-Vigorous Physical Activity From Accelerometer Data: Systematic Scoping Review
Source: Interact J Med Res. 2026 Jan 8;15:e76601. doi: 10.2196/76601 (PMC12828321; doi:10.2196/76601)
Supplement: Multimedia Appendix 2 [file ijmr_v15i1e76601_app2.docx]

Multimedia Appendix 2. The full summary of included studies (N=40 studies, ranked by health condition and alphabets of author names).

| **Reference** | **Country** | **Age mean (SD)** | **Sensor brand/ model** | **Sampling rate (Hz)** | **MVPA classification technique** | **Freature Choosing Strategy** | **Window length (s)** | **Features Selected** | **F1-score (%)**  **(MVPA)** | **Accuracy (%)**  **(MVPA)** |
| --- | --- | --- | --- | --- | --- | --- | --- | --- | --- | --- |
| **Healthy state** | | | | | | | | | | |
| Ahmadi et al., 2020 [1] | Australia | 4±0.9 | ActiGraph GT3X + | 100 | RF, SVM | handcrafted | 15 | 18 time, frequency domain features | -- | RF: wrist 14.8-83.6  hip 10.4-74.5  SVM: wrist 12.3-82.6  hip 8.9-73 |
| Ahmadi et al., 2020 [2] | Australia | 4±0.9 | ActiGraph GT3X + | 100 | RF | handcrafted | 1, 5, 10, 15 | 18 base features (time, frequency domain) & base plus temporal | wrist 69.0-82.4  hip 78.5-82.6  wrist & hip 78-86.8 | -- |
| Ahmadi et al., 2020 [3] | Australia | 13.9±3 | ActiGraph GT3X + | 30 | RF | handcrafted | 10 | 22 time, frequency domain features | 98.9 ^a^ | 98.7 ^a^ |
| Ahmadi et al., 2022 [4] | Australia | 4±0.9 | ActiGraph GT3X + | 100 | RF | handcrafted | 15 | 25 time, frequency domain features | -- | All: wrist 67.6  hip 78.2 |
| Ahmadi et al., 2023 [5] | USA & Australia | 55.8 ±12.4 | MEMS  ActiGraph GT9X | 100 | RF | handcrafted | 10 | NR | 92.0 ^a^ | 93.3 ^a^ |
| Ahmadi et al., 2023 [5] | USA & Australia | 55.8 ±12.4 | MEMS  ActiGraph GT9X | 100 | RF | handcrafted | 10 | NR | 93.7 | 93.8 |
| Ahmadi et al., 2023 [5] | UK | 18-91 | Axivity AX3 | 100 | RF | handcrafted | 10 | NR | -- | -- |
| Andò et al., 2024 [6] | Italy | -- | LSM9DS1 | 100 | K-NN, RF | handcrafted | 10 | mean and variance of raw data | 99 ^a^ | 98.1 ^a^ |
| Bai et al., 2022 [7] | USA | 72.4±7.1 | ActiGraph GT3X-BT | 100 | XGBoost | handcrafted | 60 | 49 time, frequency domain features | 86.9 | -- |
| Barua et al., 2024 [8] | Canada | 29  (18-56) | Samsung Galaxy S7 | 30 | 1D-CNN-LSTM | autonomous | 2.17 | Orientation-invariant metrics | pocket & backpack: 81, hand: 82 | -- |
| Chen et al., 2024 [9] | China | 12.3±1.0 | smartwatch "mumu" | 2 | SVM | handcrafted | 60 | mean, variance, and count of steps | -- | 89 |
| Chen et al., 2024 [9] | China | 24.9±2.6 | smartwatch "mumu" | 2 | SVM | handcrafted | 60 | mean, variance, and count of steps | -- | 88 |
| Davoudi et al., 2019 [10] | USA | 55.2±17.8 | ActiGraph GT3X+, Samsung smartwatch | 100 | RF | handcrafted | 1, 2, 4, 8, 15, 16 | 15 time, frequency domain features | RF (highest) 83.3 | RF (highest) 87.3 |
| Doherty et al., 2018 [11] | UK | 18-91 | Axivity AX3 | 100 | RF | handcrafted | 30 | 126 time, frequency domain features | 80 ^a^ | 91.4 ^a^ |
| Ellingson et al., 2016 [12] | USA | 23.9±5.3 | ActiGraph GT3X+  actiPAL | 100, 20 | ANN, DT | handcrafted | 30 | NR | SOJ-hip: 83.3 ^a^  SIP-thigh: 85.2 ^a^ | SOJ-hip: 86.3 ^a^  SIP-thigh: 87.6 ^a^ |
| Ellingson et al., 2017 [13] | USA | 23.5±4.6 | ActiGraph GT3X+ | 100 | RF | handcrafted | 15 | VM, SD, mean acceleration angle in 15s windows | 64.8 | 71.7 |
| Farrahi et al., 2020 [14] | Finland  USA  German  NR | 27.5 ±11.2  13.7±3.1  27.2±3.3  20-30 | Hookie AM20, Actigraph GT3X+, Colibri IMU, Xsens MTx IMU | 100  30  100  25 | ANN | handcrafted | 60 | 25 time, frequency domain features | -- | hip 84.9-96.6  wrist 92.7-95.6 |
| Farrahi et al., 2023 [15] | UK | 18-91 | Axivity AX3 | 100 | BiLSTM  RF  ANN  SVM  DT  NB | autonomous | 30 | 120 deep features from signal images | -- | BiLSTM: 94.7  RF: 59.7  ANN: 76.6  SVM: 82.2  DT: 71.7  NB: 64.5 |
| Freedson et al., 2011 [16] | USA | 38±12.4 | ActiGraph GT1M | 1 | ANN | handcrafted | 60 | 10 time, frequency domain features | 91 ^a^ | 81.2 ^a^ |
| **Reference** | **Country** | **Age mean (SD)** | **Sensor brand/ model** | **Sampling rate (Hz)** | **MVPA classification technique** | **Freature Choosing Strategy** | **Window length (s)** | **Features Selected** | **F1-score (%)**  **(MVPA)** | **Accuracy (%)**  **(MVPA)** |
| **Healthy state** | | | | | | | | | | |
| Hagenbuchner et al., 2015 [17] | Australia | 4.8±0.9 | ActiGraph GT3X + | 100 | ANN | handcrafted | 10, 30, 60 | 10 time, frequency domain features | SOM ^a^: 10s 41.7, 30s 57.7, 60s 58.3  MLP ^a^: 10s 57.1, 30s 28.6, 60s 71.8  DLEN ^a^: 10s 80.4, 30s 84.1, 60s 83 | SOM ^a^: 10s 57.6, 30s 68.9, 60s 67.4  MLP ^a^: 10s 70.5, 30s 54.5, 60s 78  DLEN ^a^: 10s 84.1, 30s 87.1, 60s 86.4 |
| Hibbing et al., 2018 [18] | USA | 9.4±2.1 | ActiGraph GT3X-BT | 100 | ANN, DT | handcrafted | 15 | 10 time, frequency domain features | hip 75.4 ^a^, wrist 70.8 ^a^ | hip 82.1 ^a^, wrist 77.2 ^a^ |
| Hibbing et al., 2018 [18] | USA | 10.0±2.2 | ActiGraph GT3X+ | 100 | ANN, DT | handcrafted | 15 | 10 time, frequency domain features | -- | ANN: hip 82.4, wrist 78.3  adapted Sojourn: hip 71.6, wrist 70.3 |
| Li et al., 2020 [19] | USA | 4.0±0.5 | ActiGraph GT3X-BT | 30 | k-means | handcrafted | 60 | VM counts per min | -- | -- |
| Mardini et al., 2021 [20] | USA | 61.7±17.7 | ActiGraph GT3X-BT | 100 | DT, RF, XGBoost, LASSO | handcrafted | 60 | 49 time, frequency domain features | DT: 82.8  RF: 87.5  LASSO: 83.6  XGBoost: 87.9 | -- |
| Montoye et al., 2016 [21] | USA | 22.0±4.2 | ActiGraph GT3X+  GENEActiv | 40, 20 | ANN | handcrafted | 30 | 15 time, frequency domain features | thigh 99.4, hip 91.7,  left wrist 94.6,  right wrist 79 | thigh 99.6, hip 94.1  left wrist 96.2, right wrist 86.7 |
| Montoye et al., 2017 [22] | USA | 22.0±4.2 | activPAL3 | 20 | ANN | handcrafted | 30 | mean, variance per axis | -- | -- |
| Montoye et al., 2021 [23] | USA | 40.8±19.2 | ActiGraph GT9X Link | 60, 30 | RF | handcrafted | 30 | 87 time, frequency domain features | hip ^a^: lab 70.1, free-living 74.4  wrist ^a^: lab 65.3, free-living 78.7 | hip: ^a^ lab 92.5, free-living 93.4  wrist ^a^: lab 91.8, free-living 94.3 |
| Nawaratne et al, 2021 [24] | Australia | 45.0±11.0 | ActiGraph GT3X + | 100 | CNN | handcrafted | 30 | CNN autonomously derives features from raw data | CNN-c: 72.5 ^a^  CNN-r: 73.4 ^a^ | CNN-c: 96.8 ^a^  CNN-r: 96.5 ^a^ |
| Nnamoko et al., 2021 [25] | UK | 69.3±8.0 | GENEActiv  ActiGraph | 60 | additive regression tree | autonomous | 1 | NR | -- | -- |
| O'Driscoll et al., 2021 [26] | UK | 44.4 ±14.1  31.9±10.2 | ActiGraph GT3-X  SenseWear Armband | 1 | RF, ANN, K-NN, SVM, Gradient Boosting | handcrafted | 60 | mean, SD, percentiles, correlations | Actigraph: 80.4-92.4 ^a^  SenseWear: 86-93.2 ^a^ | Actigraph: 71.9-90.9 ^a^  SenseWear: 81.9-92 ^a^ |
| Pober et al., 2006 [27] | USA | 24.8±4.2 | Actigraph MTI 7164 | 1 | QDA  HMM | handcrafted | 15 | mean, (co)variance of counts | QDA: 100 ^a^  HMM: 100 ^a^ | QDA: 99.9 ^a^  HMM: 99.8 ^a^ |
| Skjødt et al., 2025 [28] | Denmark | 80.2±3.7 | ActiGraph GT3X +  GENEActiv  Axivity AX3 | 100 | RF | handcrafted | 60 | ENMO, mean and SD of 3 axes, vector, inclination and angel | hips: 34 ^m^-74 ^v^  wrists: 40 ^m^ -79 ^v^  thigh: 43 ^m^ -75 ^v^  lower back: 37 ^m^ -71 ^v^ | -- |
| Staudenmayer et al., 2009 [29] | USA | 35  (21-69) | Actigraph model 7164 | 10 | ANN | handcrafted | 60 | 10 time, frequency domain features | 95.2 ^a^ | 98.9 ^a^ |
| Staudenmayer et al., 2015 [30] | USA | 24.1  (20-39) | ActiGraph GT3X+ | 80 | RF, DT | handcrafted | 15 | mean VM, SD, spectral power, dominant frequency, angle of acceleration | RF: 91.3 ^a^  DT: 91.9 ^a^ | RF: 87.1 ^a^  DT: 87.9 ^a^ |
| Trost et al., 2012 [31] | USA | 11±2.7 | ActiGraph GT1M | 30 | ANN | handcrafted | 10, 60 | 10 time, frequency domain features | 10s 93.0 ^a^  60s 93.4 ^a^ | 10s 93.0 ^a^  60s 93.5 ^a^ |
| Trost et al., 2012 [31] | USA | 11±2.7 | ActiGraph GT1M | 30 | ANN | handcrafted | 60 | 10 time, frequency domain features | SOM ^a^ 86.8  MLP ^a^ 97.7  DLEN ^a^ 98.7 | SOM ^a^ 88.1  MLP ^a^ 97.7  DLEN ^a^ 98.7 |
| **Reference** | **Country** | **Age mean (SD)** | **Sensor brand/ model** | **Sampling rate (Hz)** | **MVPA classification technique** | **Freature Choosing Strategy** | **Window length (s)** | **Features Selected** | **F1-score (%)**  **(MVPA)** | **Accuracy (%)**  **(MVPA)** |
| **Healthy state** | | | | | | | | | | |
| Trost et al., 2018 [32] | Australia | 4.8±0.9 | ActiGraph GT3X + | 100 | RF, SVM | handcrafted | 15s | 18 time, frequency domain features | RF ^a^: hip 87.5  wrist 86.9  hip+wrist 87.5  SVM ^a^: hip 87.5  wrist 88.5  hip+wrist 89.7 | RF ^a^: hip 86.1  wrist 85.2  hip+wrist 86  SVM ^a^ : hip 86.2  wrist 87.1  hip+wrist 88.4 |
| Tsanas, 2025 [33] | UK | 18-91 | Axivity AX3 | 100 | RF, HMM | handcrafted | 60 | 44 raw, smoothened features | 62 ^a^ | 95.4 ^a^ |
| Walmsley et al., 2021 [34] | UK | 18-91 | Axivity AX3 | 100 | RF, HMM | handcrafted | 30 | 50 time, frequency domain features | 85 ^a^ | 92.5 ^a^ |
| Wang et al., 2025 [35] | UK | 18-91 | Axivity AX3 | 100 | ViT-BiLSTM, CNN-BiLSTM, ViT, CNN, BiLSTM | autonomous | 1, 5, 10, 15, 30 | NR | at 10 epoch ^a^:  ViT-BiLSTM (Gravity) 99.6  ViT-BiLSTM (METs) 97.1  CNN-BiLSTM 91.4 ViT 79.8  CNN 70.3  BiLSTM 73.6 | at 10 epoch ^a^:  ViT-BiLSTM (Gravity) 99.9  ViT-BiLSTM (METs) 98.1  CNN-BiLSTM 97.7 ViT 95  CNN 91.9  BiLSTM 92.4  1-30s: 97-99 |
| Wullems et al., 2017 [36] | UK | 73.5±6.3 | GENEActiv | 60 | RF | handcrafted | 10 | 20 time, frequency domain features | 95.3 ^a^ | 95.1 ^a^ |
| Wullems et al., 2024 [37] | UK | 70.0±12.0 | GENEActiv | 60 | RF | handcrafted | 10 | NR | 80.9 ^a^ | 84.7 ^a^ |
| Zhou et al., 2021 [38] | China | 5.0±0.9 | Custom IMU sensor | 50 | BiLSTM | handcrafted | 5.12 | 48 time,frequency domain features | lower MPA 61, higher MPA 33, VPA 66 | lower MPA 53, higher MPA 30,  VPA 78 |
| **Clinical conditions** | | | | | | | | | | |
| Bianchim et al., 2024 [39] | UK | 12.0±2.8 | GENEActiv  ActiGraph | 100 | K-NN, RF, XGBoost | handcrafted | 1.5 | 9 time domain features | All: 100 | All: 99.5-100 |
| Cescon et al., 2021 [40] | USA | 44.9±5.0 | Empatica E4 wristband | 32 | RF | handcrafted | 1 | 66 time, frequency domain features | 90 | -- |

***Note***: ^a^ Metrics (F1-score and accuracy) calculated from confusion matrix provided by the original article, ^m^ means the values of moderate physical activity, ^v^ means the values of vigorous physical activity.

“--” and “NR” indicates “Not Reported”. ANN = artificial neural network, BiLSTM = bidirectional long–short-term memory, CNN = convolutional neural network, CNN-c = physical activity intensity category as classified by CNN, CNN-r = energy expenditure predicted by CNN, DLEN = the deep learning ensemble network, DT = decision tree, ENMO = Euclidean norm minus one, HMM = hidden Markov model, LSTM = long–short-term memory, k-means = k-means cluster analysis, K-NN = k-nearest neighbor, LASSO = least absolute shrinkage and selection operator, MLP = multi-layer perceptron network, MVPA=moderate-to-vigorous physical activity, NB = Naïve Bayes, NR = not reported; QDA = quadratic discriminant analysis, RF = random forest, SD = standard deviation, SIP = method proposed by Ellingson (2016) [12], SOJ = Lyden’s sojourn method [41], combining artificial neural network and decision tree, SOM = the self-organizing map, SVM = support vector machine, ViT = vision transformer, VM = vector magnitude; XGBoost = eXtreme Gradient Boosting.

**The ground truth method**: DO = direct observation, IC = indirect calorimetry, PRE = predefined activity schedule.

**The health condition**: CF = cystic fibrosis, T1D = type 1 diabetes

## References

1. Ahmadi MN, Brookes D, Chowdhury A, Pavey T, Trost SG. Free-living Evaluation of Laboratory-based Activity Classifiers in Preschoolers. Medicine and science in sports and exercise. United States2020. p. 1227-34.

2. Ahmadi MN, Pavey TG, Trost SG. Machine Learning Models for Classifying Physical Activity in Free-Living Preschool Children. Sensors [Internet]. 2020; 20(16).

3. Ahmadi MN, Pfeiffer KA, Trost SG. Physical Activity Classification in Youth Using Raw Accelerometer Data from the Hip. MEASUREMENT IN PHYSICAL EDUCATION AND EXERCISE SCIENCE. 2020 APR 2;24(2):129-36. doi: 10.1080/1091367X.2020.1716768.

4. Ahmadi MN, Trost SG. Device-based measurement of physical activity in pre-schoolers: Comparison of machine learning and cut point methods. PloS one. United States: Public Library of Science; 2022. p. e0266970.

5. Ahmadi MN, Hamer M, Gill JMR, Murphy M, Sanders JP, Doherty A, et al. Brief bouts of device-measured intermittent lifestyle physical activity and its association with major adverse cardiovascular events and mortality in people who do not exercise: a prospective cohort study. Lancet Public health. England: Elsevier, Ltd; 2023. p. e800-e10.

6. Andò B, Manenti M, Greco D, Pistorio A, editors. An Embedded Sensing Methodology for the Classification of Activity Rate. 2024 IEEE International Conference on Metrology for eXtended Reality, Artificial Intelligence and Neural Engineering (MetroXRAINE); 2024 21-23 Oct. 2024.

7. Bai C, Wanigatunga AA, Saldana S, Casanova R, Manini TM, Mardini MT. Are Machine Learning Models on Wrist Accelerometry Robust against Differences in Physical Performance among Older Adults? Sensors (Basel, Switzerland). Switzerland: MDPI; 2022.

8. Barua A, Jiang X, Fuller D. The effectiveness of simple heuristic features in sensor orientation and placement problems in human activity recognition using a single smartphone accelerometer. BioMed Eng Online. 2024 Feb;23(1). PMID: 38368358. doi: 10.1186/s12938-024-01213-3.

9. Chen D, Du Y, Liu Y, Hong J, Yin X, Zhu Z, et al. Development and validation of a smartwatch algorithm for differentiating physical activity intensity in health monitoring. Sci Rep. 2024;14:9530. PMID: 38664457. doi: 10.1038/s41598-024-59602-6.

10. Davoudi A, Wanigatunga AA, Kheirkhahan M, Corbett DB, Mendoza T, Battula M, et al. Accuracy of Samsung Gear S Smartwatch for Activity Recognition: Validation Study. JMIR MHEALTH AND UHEALTH. 2019 Feb 6;7(2). PMID: 30724739. doi: 10.2196/11270.

11. Doherty A, Smith-Byrne K, Ferreira T, Holmes MV, Holmes C, Pulit SL, et al. GWAS identifies 14 loci for device-measured physical activity and sleep duration. Nature Communications. 2018 2018/12/10;9(1):5257. doi: 10.1038/s41467-018-07743-4.

12. Ellingson LD, Schwabacher IJ, Kim Y, Welk GJ, Cook DB. Validity of an Integrative Method for Processing Physical Activity Data. Medicine and science in sports and exercise. United States2016. p. 1629-38.

13. Ellingson LD, Hibbing PR, Kim Y, Frey-Law LA, Saint-Maurice PF, Welk GJ. Lab-based validation of different data processing methods for wrist-worn ActiGraph accelerometers in young adults. Physiological measurement. England: IOP Pub. Ltd; 2017. p. 1045-60.

14. Farrahi V, Niemelä M, Tjurin P, Kangas M, Korpelainen R, Jämsä T. Evaluating and Enhancing the Generalization Performance of Machine Learning Models for Physical Activity Intensity Prediction From Raw Acceleration Data. IEEE Journal of Biomedical and Health Informatics. 2020;24(1):27-38. doi: 10.1109/JBHI.2019.2917565.

15. Farrahi V, Muhammad U, Rostami M, Oussalah M. AccNet24: A deep learning framework for classifying 24-hour activity behaviours from wrist-worn accelerometer data under free-living environments. International journal of medical informatics. Ireland: Elsevier Science Ireland Ltd; 2023. p. 105004.

16. Freedson PS, Lyden K, Kozey-Keadle S, Staudenmayer J. Evaluation of artificial neural network algorithms for predicting METs and activity type from accelerometer data: validation on an independent sample. Journal of applied physiology (Bethesda, Md : 1985). United States: American Physiological Society; 2011. p. 1804-12.

17. Hagenbuchner M, Cliff DP, Trost SG, Nguyen Van T, Peoples GE. Prediction of activity type in preschool children using machine learning techniques. J Sci Med Sport. 2015 Jul;18(4):426-31. PMID: 25088983. doi: 10.1016/j.jsams.2014.06.003.

18. Hibbing PR, Ellingson LD, Dixon PM, Welk GJ. Adapted Sojourn Models to Estimate Activity Intensity in Youth: A Suite of Tools. Med Sci Sports Exerc. 2018 Apr;50(4):846-54. PMID: 29135657. doi: 10.1249/MSS.0000000000001486.

19. Li S, Howard JT, Sosa ET, Cordova A, Parra-Medina D, Yin Z. Calibrating Wrist-Worn Accelerometers for Physical Activity Assessment in Preschoolers: Machine Learning Approaches. JMIR formative research. 2020;4:e16727. PMID: 32667893. doi: 10.2196/16727.

20. Mardini MT, Bai C, Wanigatunga AA, Saldana S, Casanova R, Manini TM. Age Differences in Estimating Physical Activity by Wrist Accelerometry Using Machine Learning. Sensors (Basel, Switzerland). Switzerland: MDPI; 2021.

21. Montoye AHK, Pivarnik JM, Mudd LM, Biswas S, Pfeiffer KA. Validation and Comparison of Accelerometers Worn on the Hip, Thigh, and Wrists for Measuring Physical Activity and Sedentary Behavior. AIMS public health. 2016;3:298-312. PMID: 29546164. doi: 10.3934/publichealth.2016.2.298.

22. Montoye AHK, Pivarnik JM, Mudd LM, Biswas S, Pfeiffer KA. Evaluation of the activPAL accelerometer for physical activity and energy expenditure estimation in a semi-structured setting. Journal of science and medicine in sport. Australia: Elsevier Australia; 2017. p. 1003-7.

23. Montoye AHK, Westgate BS, Clevenger KA, Pfeiffer KA, Vondrasek JD, Fonley MR, et al. Individual versus Group Calibration of Machine Learning Models for Physical Activity Assessment Using Body-Worn Accelerometers. Medicine and science in sports and exercise. United States2021. p. 2691-701.

24. Nawaratne R, Alahakoon D, De Silva D, O'Halloran PD, Montoye AH, Staley K, et al. Deep Learning to Predict Energy Expenditure and Activity Intensity in Free Living Conditions using Wrist-specific Accelerometry. Journal of sports sciences. England: Routledge; 2021. p. 683-90.

25. Nnamoko N, Cabrera-Diego LA, Campbell D, Sanders G, Fairclough SJ, Korkontzelos I. Personalised Accelerometer Cut-point Prediction for Older Adults' Movement Behaviours using a Machine Learning approach. Computer methods and programs in biomedicine. Ireland: Elsevier Scientific Publishers; 2021. p. 106165.

26. O'Driscoll R, Turicchi J, Hopkins M, Duarte C, Horgan GW, Finlayson G, et al. Comparison of the Validity and Generalizability of Machine Learning Algorithms for the Prediction of Energy Expenditure: Validation Study. JMIR mHealth and uHealth. Canada: JMIR Publications Inc; 2021. p. e23938.

27. Pober DM, Staudenmayer J, Raphael C, Freedson PS. Development of Novel Techniques to Classify Physical Activity Mode Using Accelerometers. Medicine & Science in Sports & Exercise. 2006;38(9). doi: 10.1249/01.mss.0000227542.43669.45.

28. Skjødt M, Brønd JC, Tully MA, Tsai L-T, Koster A, Visser M, et al. Moderate and Vigorous Physical Activity Intensity Cut-Points for Hip-, Wrist-, Thigh-, and Lower Back Worn Accelerometer in Very Old Adults. Scandinavian Journal of Medicine & Science in Sports. Denmark: Munksgaard International Publishers; 2025. p. e70009.

29. Staudenmayer J, Pober D, Crouter S, Bassett D, Freedson P. An artificial neural network to estimate physical activity energy expenditure and identify physical activity type from an accelerometer. Journal of applied physiology (Bethesda, Md : 1985). United States: American Physiological Society; 2009. p. 1300-7.

30. Staudenmayer J, He S, Hickey A, Sasaki J, Freedson P. Methods to estimate aspects of physical activity and sedentary behavior from high-frequency wrist accelerometer measurements. Journal of applied physiology (Bethesda, Md : 1985). United States: American Physiological Society; 2015. p. 396-403.

31. Trost SG, Wong W-K, Pfeiffer KA, Zheng Y. Artificial neural networks to predict activity type and energy expenditure in youth. Med Sci Sports Exerc. 2012;44:1801-9. PMID: 22525766. doi: 10.1249/MSS.0b013e318258ac11.

32. Trost SG, Cliff DP, Ahmadi MN, Nguyen Van T, Hagenbuchner M. Sensor-enabled Activity Class Recognition in Preschoolers: Hip versus Wrist Data. Med Sci Sports Exerc. 2018 Mar;50(3):634-41. PMID: 29059107. doi: 10.1249/MSS.0000000000001460.

33. Tsanas A. Accurately Inferring Physical Activity Levels and Sleep From Wrist-Worn Actigraphy Recordings With Sample Rates as Low as 10 Hz. IEEE Access. 2025;13:27257-67. doi: 10.1109/ACCESS.2025.3539278.

34. Walmsley R, Chan S, Smith-Byrne K, Ramakrishnan R, Woodward M, Rahimi K, et al. Reallocation of time between device-measured movement behaviours and risk of incident cardiovascular disease. Br J Sports Med. 2021. PMID: 34489241. doi: 10.1136/bjsports-2021-104050.

35. Wang L, Luo Z, Zhang T. A novel ViT-BILSTM model for physical activity intensity classification in adults using gravity-based acceleration. Biomed Eng. 2025 Feb;7(1):2-. PMID: 39891283. doi: 10.1186/s42490-025-00088-2.

36. Wullems JA, Verschueren SMP, Degens H, Morse CI, Onambélé GL. Performance of thigh-mounted triaxial accelerometer algorithms in objective quantification of sedentary behaviour and physical activity in older adults. PloS one. United States: Public Library of Science; 2017. p. e0188215.

37. Wullems JA, Verschueren SMP, Degens H, Morse CI, Onambele-Pearson GL. Concurrent Validity of Four Activity Monitors in Older Adults. SENSORS. 2024 Feb;24(3). PMID: 38339613. doi: 10.3390/s24030895.

38. Zhou L, Qu X, Zhang T, Wu J, Yin H, Guan H, et al. Prediction of pediatric activity intensity with wearable sensors and bi-directional LSTM models. Pattern Recognit Lett. 2021 Dec;152:166-71. doi: 10.1016/j.patrec.2021.08.030.

39. Bianchim MS, McNarry MA, Barker AR, Williams CA, Denford S, Thia L, et al. A Machine Learning Approach for Physical Activity Recognition in Cystic Fibrosis. Meas Phys Educ Exerc Sci. 2024 Apr;28(2):172-81. doi: 10.1080/1091367X.2023.2271444.

40. Cescon M, Choudhary D, Pinsker JE, Dadlani V, Church MM, Kudva YC, et al. Activity detection and classification from wristband accelerometer data collected on people with type 1 diabetes in free-living conditions. Computers in Biology and Medicine. 2021 2021/08/01/;135:104633. doi: <https://doi.org/10.1016/j.compbiomed.2021.104633>.

41. Lyden K, Keadle SK, Staudenmayer J, Freedson PS. A method to estimate free-living active and sedentary behavior from an accelerometer. Med Sci Sports Exerc. 2014;46:386-97. doi: 10.1249/MSS.0b013e3182a42a2d.
